# Resilient and Sensitive Key Points of the Photosynthetic Machinery of *Coffea* spp. to the Single and Superimposed Exposure to Severe Drought and Heat Stresses

**DOI:** 10.3389/fpls.2020.01049

**Published:** 2020-07-09

**Authors:** Danielly Dubberstein, Fernando C. Lidon, Ana P. Rodrigues, José N. Semedo, Isabel Marques, Weverton P. Rodrigues, Duarte Gouveia, Jean Armengaud, Magda C. Semedo, Sónia Martins, Maria C. Simões-Costa, I. Moura, Isabel P. Pais, Paula Scotti-Campos, Fábio L. Partelli, Eliemar Campostrini, Ana I. Ribeiro-Barros, Fábio M. DaMatta, José C. Ramalho

**Affiliations:** ^1^ PlantStress & Biodiversity Lab, Centro de Estudos Florestais (CEF), Dept. Recursos Naturais, Ambiente e Território (DRAT), Instituto Superior de Agronomia (ISA), Universidade de Lisboa (ULisboa), Lisbon, Portugal; ^2^ Centro Univ. Norte do Espírito Santo (CEUNES), Dept. Ciências Agrárias e Biológicas (DCAB), Univ. Federal Espírito Santo (UFES), São Mateus, Brazil; ^3^ Unidade de Geobiociências, Geoengenharias e Geotecnologias (GeoBioTec), Faculdade de Ciências e Tecnologia (FCT), Universidade NOVA de Lisboa (UNL), Caparica, Portugal; ^4^ Unid. Investigação em Biotecnologia e Recursos Genéticos, Instituto Nacional de Investigação Agrária e Veterinária, I.P. (INIAV), Oeiras, Portugal; ^5^ Setor Fisiologia Vegetal, Centro de Ciências e Tecnologias Agropecuárias, Univ. Estadual Norte Fluminense (UENF), Darcy Ribeiro, Brazil; ^6^ Centro de Ciências Agrárias, Naturais e Letras, Universidade Estadual da Região Tocantina do Maranhão, Estreito, Brazil; ^7^ Laboratoire Innovations technologiques pour la Détection et le Diagnostic (Li2D), Service de Pharmacologie et Immunoanalyse (SPI), CEA, INRA, Bagnols-sur-Cèze, France; ^8^ Área Departamental de Engenharia Química, Instituto Superior de Engenharia de Lisboa, Instituto Politécnico de Lisboa, Lisboa, Portugal; ^9^ Dept. Biologia Vegetal, Univ. Federal Viçosa (UFV), Viçosa, Brazil

**Keywords:** acclimation, climate change, coffee, drought, heat, photoinhibition, photosynthesis

## Abstract

This study unveils the single and combined drought and heat impacts on the photosynthetic performance of *Coffea arabica* cv. Icatu and *C. canephora* cv. Conilon Clone 153 (CL153). Well-watered (WW) potted plants were gradually submitted to severe water deficit (SWD) along 20 days under adequate temperature (25/20°C, day/night), and thereafter exposed to a gradual temperature rise up to 42/30°C, followed by a 14-day water and temperature recovery. Single drought affected all gas exchanges (including *A_max_*) and most fluorescence parameters in both genotypes. However, Icatu maintained *F_v_/F_m_* and RuBisCO activity, and reinforced electron transport rates, carrier contents, and proton gradient regulation (PGR5) and chloroplast NADH dehydrogenase-like (NDH) complex proteins abundance. This suggested negligible non-stomatal limitations of photosynthesis that were accompanied by a triggering of protective cyclic electron transport (CEF) involving both photosystems (PSs). These findings contrasted with declines in RuBisCO and PSs activities, and cytochromes (*b_559_*, *f*, *b_563_*) contents in CL153. Remarkable heat tolerance in potential photosynthetic functioning was detected in WW plants of both genotypes (up to 37/28°C or 39/30°C), likely associated with CEF in Icatu. Yet, at 42/30°C the tolerance limit was exceeded. Reduced *A_max_* and increased *C_i_* values reflected non-stomatal limitations of photosynthesis, agreeing with impairments in energy capture (*F_0_* rise), PSII photochemical efficiency, and RuBisCO and Ru5PK activities. In contrast to PSs activities and electron carrier contents, enzyme activities were highly heat sensitive. Until 37/28°C, stresses interaction was largely absent, and drought played the major role in constraining photosynthesis functioning. Harsher conditions (SWD, 42/30°C) exacerbated impairments to PSs, enzymes, and electron carriers, but uncontrolled energy dissipation was mitigated by photoprotective mechanisms. Most parameters recovered fully between 4 and 14 days after stress relief in both genotypes, although some aftereffects persisted in SWD plants. Icatu was more drought tolerant, with WW and SWD plants usually showing a faster and/or greater recovery than CL153. Heat affected both genotypes mostly at 42/30°C, especially in SWD and Icatu plants. Overall, photochemical components were highly tolerant to heat and to stress interaction in contrast to enzymes that deserve special attention by breeding programs to increase coffee sustainability in climate change scenarios.

## Introduction

The global CO_2_ emissions have been increased steadily from the industrial revolution onwards, from around 280 µL CO_2_ L^-^¹ until a global atmosphere average of 407.4 in 2018 (NOAA, 2019). Further increases might lead to estimated values between 730 and 1,020 µL CO_2_ L^-^¹ in 2100 accompanied by a global warming up to 4.8°C (IPCC, 2014). In addition, heat waves and altered inter- and intra-annual precipitation patterns, with periods of prolonged drought to extreme rainfall events, have also been predicted (IPCC, 2014; IPCC, 2018).

Heat and drought stresses, which are major environmental constraints to plant growth and crop productivity, have been associated with decreases in both stomatal conductance (*g*_s_) and net CO_2_ assimilation rates (*P*_n_) (Long et al., 2006). These decreases predispose leaves to photoinhibitory damage due to decreased energy use through photosynthesis (Baker and Rosenqvist, 2004; Lambers et al., 2008; Haworth et al., 2018), which can be exacerbated when these stresses are superimposed. As a consequence, growth and productivity of agricultural crops are depressed under these conditions to a greater extent than to each single applied stress (Sehgal et al., 2017; Urban et al., 2018). Additionally, the type and magnitude of plant responses to combined stresses usually differ from the responses triggered by single stress exposure (Mittler, 2006; Sehgal et al., 2017). This might be even more relevant for perennial crops such as coffee, which can have a lifespan up to 30 years (Bunn et al., 2015) or even more.

In particular, drought is the major threat to world agricultural production. Water shortage reduces plant growth, nutrient uptake, photosynthesis, and assimilate partitioning, therefore strongly reducing crop yields (Fahad et al., 2017; Lamaoui et al., 2018). Photosynthesis is a major primary process affected by water constraints (Chaves et al., 2009). Under mild drought, the photosynthetic decline is mostly related to stomatal closure that avoids additional water loss through transpiration. However this also reduces the CO_2_ supply to ribulose-1,5-bisphosphate carboxylase/oxygenase (RuBisCO), although with limited impact on photochemistry and photosynthetic capacity (*A*_max_). Under more prolonged/severe water constraint, non-stomatal limitations will progressively occur with impairments to several photosynthetic components, namely in pigment pools, photosystems (PSs) functioning, and activity of key enzymes such as RuBisCO (Chaves et al., 2003; Ramalho et al., 2014a; Fahad et al., 2017). Limited photochemical energy use can additionally impose a secondary stress related to oxidative conditions due to the uncontrolled generation of reactive species of oxygen (ROS) and chlorophyll, leading to damages to the photosynthetic apparatus (*e.g*., D1 protein, lipids, electron transport) (Chaves et al., 2009; Osakabe et al., 2014). Therefore, at the cellular level drought tolerance is often associated with the triggering of photoprotective and antioxidative mechanisms. These include cyclic electron flow (CEF) around PSs and antioxidative molecules (*e.g.*, enzymes, carotenoids) (Chu and Chiu, 2016; Ramalho et al., 2018b; Sun et al., 2018).

High temperature can also cause physiological, biochemical and molecular disturbances, affecting all major physiological processes (Long et al., 2006). As regards to the photosynthetic pathway, heat modifies pigment composition (Hasanuzzaman et al., 2013) while reducing electron transport and RuBisCO activity (Crafts-Brandner and Salvucci, 2000; Haldimann and Feller, 2004). Additionally, under heat the chemical bonds are weakened and membrane lipid bilayer becomes more fluid and destabilized (Wahid et al., 2007; Los and Zinchenko, 2009), thus compromising membrane-based events such as chloroplast light energy capture and electron transport (Scotti-Campos et al., 2019). High temperature further alters gas diffusion throughout the mesophyll (Lambers et al., 2008), and stimulates respiration and photorespiration more than photosynthesis. Finally, heat stress alters hormones and primary and secondary metabolite balance (Wahid et al., 2007), causes protein denaturation and aggregation as well as ROS overproduction and inhibition of transcription and translation (Wahid et al., 2007; Song et al., 2014; Dusenge et al., 2019), thereby further concurring to depress plant growth and crop yields.

Coffee chain of value is supported by *Coffea. arabica* L. and *Coffea canephora* Pierre ex A. Froehner species, which are responsible for nearly 99% of the global coffee production. Coffee is one of the world’s most traded agricultural products, being cropped in approximately 80 tropical countries. It supports the livelihoods of *ca*. 25 million smallholder farmers and *ca*. 100-125 million people in its worldwide chain of value (Osorio, 2002; Semedo et al., 2018; Ramalho et al., 2018a; DaMatta et al., 2019).

The estimated increase of air [CO_2_] in the coming years could have a positive impact on mineral balance (Martins et al., 2014), C-assimilation (Rodrigues et al., 2016) and even on productivity (DaMatta et al., 2019), helping the preservation of coffee bean quality under supra-optimal temperatures (Ramalho et al., 2018a) if sufficient water is available. However, a growing concern is related to this crop sustainability given that coffee is cultivated in tropical areas, which are predicted to be strongly impacted by climate change (IPCC, 2018). Indeed, inadequate temperature and water availability are known as the most important environmental constraints for the coffee crop (DaMatta et al., 2018; Ramalho et al., 2018b). In addition, coffee is extensively cropped under full sunlight exposure what can further exacerbate both temperature and drought stresses. Climate change, associated with a greater frequency of extreme events of temperature and drought, is expected to reduce crop yield and sustainability, with likely greater impacts in *C. arabica* that is considered more sensitive to heat than *C. canephora* (DaMatta and Ramalho, 2006; DaMatta et al., 2018). Furthermore, predicted future warming may cause the extinction of at least 60% of all coffee species (Davis et al., 2019).

Coffee plants have a common set of response mechanisms allowing them to cope with single stress events, including high irradiance, drought, cold, and heat (Nunes et al., 1993; Pinheiro et al., 2004; Ramalho et al., 2014b; Martins et al., 2019). Under water shortage, drought-tolerant coffee genotypes reduce *g*_s_ to avoid excessive transpiration and trigger antioxidant molecules (DaMatta et al., 2003; Pinheiro et al., 2004; Dias et al., 2007). Regarding heat stress, recent studies showed that coffee can maintain photosynthetic performance up to 37°C, well above what was traditionally assumed (DaMatta et al., 2018). Such tolerance has been shown to be supported by the reinforcement of the antioxidant system (Martins et al., 2016; Rodrigues et al., 2016), and adjustments in the lipid profile of chloroplast membranes (Scotti-Campos et al., 2019). However, the predicted scenarios of climate changes point to future greater exposure to the combination of heat and drought stresses. Therefore, the understanding of the underlying mechanisms by which the coffee plant deals with these single and superimposed stresses is of utmost importance for future crop sustainability. In this context, we conducted an in-depth analysis of plant impacts and responses to drought, heat and their interaction on the photosynthetic performance. Morphological (stomata traits), physiological (gas exchanges, chlorophyll *a* fluorescence), and biochemical (thylakoid electron transport and carriers, enzyme activities, and proteins involved in CEF) traits were evaluated. Our findings provide paramount evidence regarding the functioning of the photosynthetic machinery under harsh heat and/or drought conditions, unveiling relevant tolerance/sensitivity points in two cropped genotypes from the most cultivated coffee species. This information should be taken into account in future adaptation/breeding efforts to ongoing climate changes and future harsher environmental scenarios.

## Material and Methods

### Plant Material and Growth Conditions

Plants of two cultivated genotypes (in Brazil), *C. canephora* Pierre ex A. Froehner cv. Conilon Clone 153 (CL153) and *C. arabica* L. cv. Icatu Vermelho (an introgressed variety resulting from a cross of *C. canephora* and *C. arabica* cv. Bourbon Vermelho, then further crossed with *C. arabica* cv. Mundo Novo), were used. Seven-year-old plants were grown in 80 L pots in walk-in growth chambers (EHHF 10000, ARALAB, Portugal) under controlled temperature (25/20°C, day/night), irradiance (*ca*. 700–800 μmol m^-2^ s^-1^), relative humidity (70%), photoperiod (12 h), and 380 μL CO_2_ L^-1^ air. Plants were maintained without restrictions in water, nutrients (see Ramalho et al., 2013a,b), or root development (as judged by visual examination at the end of the experiment after removing the plants from their pots).

### Drought and Temperature Stress Implementation

Water and heat stresses were imposed gradually in order to allow plant acclimation. Initially, watering treatments were established under adequate temperature (25/20°C, day/night), corresponding to control well-watered (WW) or severe water deficit (SWD) conditions, which represented approximately 80 or 10% of maximal pot water availability, respectively (Ramalho et al., 2018b). Drought conditions were imposed along two weeks by partially withholding irrigation (through a partial reposition of water that was lost in each pot) until stability of predawn water potential (*Ψ_pd_*) at values below −3.0 MPa for SWD plants, whereas WW plants were maintained at *Ψ_pd_* above −0.35 MPa. Water availability conditions were thereafter kept for another five days before the onset of temperature increase.

The temperature rise from 25/20°C up to 42/30°C was imposed on both WW and SWD plants at a rate of 0.5°C day^-1^ (diurnal temperature) with 5 days of stabilization at 31/25, 37/28, and 42/30°C to allow for programmed evaluations. Afterwards, the temperature was readjusted to 25/20°C and the plants from both water treatments were fully irrigated and then monitored over a recovery period of 14 days (Rec14). Overall, the

SWD plants reached the desired *Ψ_pd_* within 14 days upon gradual drought imposition, and were maintained at this condition plus 54 days,(49 of which during the temperature rise from 25/20°C to 42/30°C). The entire experiment lasted 82 days.

Determinations were performed on newly matured leaves from the upper third part of 6-8 plants per treatment, usually at 25/20, 31/25, 37/28, 42/30°C, and at Rec14. Additionally, some non-destructive parameters were further monitored at the intermediate temperatures of 28/23, 34/28, 39/30°C, and after 4 days (Rec4) or 7 to 10 (Rec7-10) days of stress relief.

Unless otherwise stated, evaluations or samplings were performed under photosynthetic steady-state conditions after *ca*. 2 h of illumination. For biochemical evaluations, leaf material was collected from 6 to 8 plants of each genotype, flash frozen in liquid nitrogen and stored at −80°C until analysis. Leaf tissue extractions were performed using an ice-cold mortar and pestle, as well as cold homogenizing solutions.

### Leaf Water Status

Leaf *Ψ_pd_* was determined at predawn immediately after leaf excision (Schölander et al., 1965) using a pressure chamber (Model 1000, PMS Instrument Co., USA). This was performed on 5–6 replicates per treatment, every two or three days, but only the data at the main temperature points for data collection (considering temperature rise, and both heat and drought recoveries) are presented.

### Stomatal Traits

Imprints from the leaf abaxial surface were taken from five plants (two leaves per plant, and three different areas per leaf) and observed under an optical light microscope (Ramalho et al., 2013b). Stomatal density (SD) was calculated as the number of stomata per unit of leaf area, and the stomatal index (SI) was calculated as SI = [(stomata)/(total cells + stomata)] x 100. Stomatal area (SA) was measured in 60 randomly selected stomata (using the same leaves) with an ocular micrometer. The area of each individual stoma was calculated as SA = πab, where *a* is 1/2 length and *b* is 1/2 width, thus assuming an elliptical stomata shape.

### Leaf Gas Exchanges

The net photosynthesis rate (*P*_n_), stomatal conductance (*g*_s_), transpiration rate (*E*) rate, and internal [CO_2_] (C_i_) were obtained using a portable open-system IRGA (Li-Cor 6400, LiCor, USA) with an external [CO_2_] supply of *ca.* 400 µL L^-1^, and *ca.* 650 µmol m^-2^ s^-1^ of irradiance. Instantaneous water use efficiency (*WUE*) was calculated as the *P*_n_ */E* ratio.

Photosynthetic capacity (*A*_max_), representing the photosynthetic rate obtained under saturating light and [CO_2_], was measured in 1.86 cm^2^ leaf discs through the evolution of O_2_ using a Clark-type O_2_ electrode (LD2/2; Hansatech, Kings Lynn, UK). *A*_max_ was obtained at 25°C, *ca*. 7% [CO_2_] (supplied by 400 μL 2 M KHCO_3_), and by exposing the leaf samples to increasing irradiance up to 1,200 μmol m^-2^ s^-1^ using a Björkman lamp (Hansatech) and neutral filters.

### Chlorophyll a Fluorescence

Chlorophyll (Chl) *a* fluorescence parameters were determined on the same leaves and conditions used for gas exchange measurements using a PAM-2000 system (H. Walz, Germany), exactly as previously described (Rodrigues et al., 2016). Measurements of the (i) minimal fluorescence from excited Chl *a* molecules from the antennae, before excitation energy migrates to the reaction centers *(F_0_)*, (ii) maximal fluorescence, corresponding to the complete reduction of primary photosystem (PS) II acceptors (*F_m_*), and (iii) maximal PSII photochemical efficiency (*F_v_/F_m_*) were performed on overnight dark-adapted leaves. *F_0_* was determined by using a weak light (< 0.5 μmol m^-2^ s^-1^) beam, while *F_m_* was obtained through a saturation pulse of 0.8 s of *ca*. 7,500 μmol m^-2^ s^-1^ of actinic light.

A second set of parameters were evaluated under photosynthetic steady-state conditions, with 650 μmol m^-2^ s^-1^ of actinic light and superimposed saturating flashes: *q_L_*, *Y_(II)_, Y_(NPQ)_, Y_(NO)_*, *F_v_’/F_m_’* (Kramer et al., 2004; Schreiber, 2004; Klughammer and Schreiber, 2008; Huang et al., 2011) and *F_s_/F_m_’* (Stirbet and Govindjee, 2011). *F_0_’*, which is required for the quenching calculations, was measured in the dark immediately after the actinic light was switched off and before the first fast phase of the fluorescence relaxation kinetics. *F_v_’/F_m_’* expresses the PSII photochemical efficiency of energy conversion under light exposure; *q_L_* is the photochemical quenching based on the concept of interconnected PSII antennae, and represents the proportion of energy captured by open PSII centers and driven to photochemical events; *F_s_/F_m_’* is a predictor of the rate constant of PSII inactivation. Estimates of photosynthetic quantum yields of non-cyclic electron transfer (*Y_(II)_*), photoprotective regulated energy dissipation of *PSII* (*Y_(NPQ)_*), and non-regulated energy dissipation (heat and fluorescence) of PSII (*Y_(NO)_*), where (*Y_(II)_+Y_(NPQ)_+Y_(NO)_*=1), were also obtained.

### Thylakoid Electron Transport Rates

The sub-chloroplast membrane fractions were obtained from a pool of leaves (*ca*. 5 g FW) from 3 plants (in triplicate), as previously described for coffee leaves (Ramalho et al., 1999). The *in vivo* electron transport rates associated with PSI (DCPIPH_2_ → MV) and PSII, including (H_2_O → DCPIP) or excluding (DPC → DCPIP) the oxygen-evolving complex (*OEC*), were obtained with an O_2_ electrode (LW2, Hansatech) using 1 mL of reaction mixture containing *ca.* 100 mg Chl, at 25°C under *ca.* 3000 μmol m^-2^ s^-1^ irradiance supplied by a Björkman lamp.

### Thylakoid Electron Carriers

Chloroplast isolation for cytochrome determinations was performed using a pool of leaves of *ca*. 7 g FW, from 3 plants (in triplicate), following the procedures of Spiller and Terry (1980) with modifications for coffee leaves (Ramalho et al., 1999). The concentrations of cytochromes (Cyt) b*_559LP_*, b*_559HP_*, b*_563_* and *f* were obtained using a spectrophotometer (UV-Vis Shimadzu UV-1800, Japan) with readings at 545 nm, with isosbest wavelengths of 528 and 568 nm for cytochrome b*_559_*, and 552 and 572 nm for cytochrome b*_563_* (Houchins and Hind, 1984), An extinction coefficient of 20 mmol L^-1^ cm^-1^ was assumed. For Cyt *f* concentration, readings were performed at 554 nm, and an extinction coefficient of 19.7 mmol L^-1^ cm^-1^ was used.

The pool of the redox form of plastoquinone (PQ-9) was determined from sub-chloroplast fractions that were obtained from a pool of leaves of *ca.* 5 g FW, from 3 plants (in triplicate), following Droppa et al. (1987) with minor modifications (Ramalho et al., 1999). PQ-9 content was determined according to Redfearn and Friend (1962) by measuring the absorption difference between the oxidized and reduced forms of PQ-9 at 255 nm, relative to isosbest wavelengths of 276 and 308 nm. An extinction coefficient of 14.8 mmol L^-1^ cm^-1^ was assumed.

### Photosynthetic Enzyme Activities

Activities of ribulose-1,5-bisphosphate carboxylase/oxygenase (RuBisCO; EC 4.1.1.39) (Osório et al., 2006; Tazoe et al., 2008) and ribulose-5-phosphate kinase (Ru5PK; EC 2.7.1.19) (Souza et al., 2005) were adapted for coffee leaves (Ramalho et al., 2003; Ramalho et al., 2013b). In detail, leaf material was finely powdered in liquid nitrogen, and an aliquot of *ca*. 100 mg FW was taken and homogenized in 1 mL extraction buffer consisting of 100 mM Tris-HCl, pH 8, containing 10 mM MgCl_2_, 15 mM NaHCO_3_, 10 mM β-mercaptoethanol, 2 mM DTT, 1% (v/v) Triton X-100, 2), 10% (v/v) glycerol and 2% (v/v) “Complete-protease inhibitor cocktail” (Roche, ref. 04693159001), together with 100 mg insoluble PVPP per homogenate. The extracts were centrifuged (16,000 *g*, 15 min, 4°C) and the obtained clean supernatant was used for spectrophotometric assessment of enzyme activities at 25°C, in a final volume of 1 mL.

*RuBisCO activities—*an assay medium containing 50 mM Tris-HCl buffer, pH 8.0, 15 mM MgCl_2_, 20 mM NaHCO_3_, 100 mM phosphocreatine, 10 mM ATP, 0.2 mM NAPH, 20 U mL^-1^ creatine kinase, 15 U mL^-1^, 3-phosphoglycerate kinase, and 15 U mL^-1^ glyceraldehyde-3-phosphate dehydrogenase was used for determination of initial and total RuBisCO activities. For the initial activity, to the assay medium were added 10 mM RuBP, and then 20 μL of the clean supernatant, followed by immediate reading. For the total activity, to the assay medium were added 20 μL of the clean supernatant, followed by a 20 min incubation period, after which the reaction was started with addition of 10 mM RuBP. In both cases measurements followed the 3-PGA-dependent NADH oxidation at 340 nm (Osório et al., 2006).

*Ru5PK activity—*the activity was determined according to the method of Souza et al. (2005). Briefly, 20 μL of clean supernatant were added to the spectrophotometer cell with 100 mM Tris-HCl pH 8.0 buffer assay, containing 8 mM MgCl_2_, 40 mM KCl, 20 mM phosphoenolpyruvate, 5 mM ATP, 1 mM NADH, 20 mM DTT, 8 U pyruvate kinase, 10 U mL^-1^ lactate dehydrogenase and 5 U mL^-1^ phosphoriboisomerase. After a 15 min incubation period, the reaction was started by adding 10 µL of 500 mM ribose-5-phosphate, and NADH oxidation was monitored at 340 nm.

### Proteins Associated With Thylakoid Cyclic Electron Flow

#### Protein Extraction and Trypsin Proteolysis

Protein extraction followed Parkhey et al. (2015) with some modifications. Briefly, *ca*. 200 mg of powdered frozen leaves were suspended in 1.5 mL of TCA (10% w/v in acetone), vortexed and incubated during 30 min at 20°C. After centrifugation (12,300 *g*, 10 min, 4°C) the supernatant was discarded. This washing step was repeated and the pellet was then mixed with 1.5 mL of 0.1 M ammonium acetate in 80% v/v methanol, and left for 30 min. The sample was centrifuged (12,300 *g*, 10 min, 4°C), the dried residue was washed once more and an additional washing step was performed with 80% v/v acetone at the same conditions. The well-dried residue was treated with 500 µL of Tris-saturated phenol pH 8.0 and 500 µL of Tris-HCl-β-mercaptoethanol-SDS buffer (0.1 M Tris-HCl, 5% v/v β-mercaptoethanol, 2% w/v SDS and 30% w/v sucrose, pH 8.0). After 1 h of incubation at room temperature, the mixture was centrifuged (12,300 *g*, 10 min, 4°C) and the upper phenolic phase was removed. To precipitate the proteins, 1.5 mL of 0.1 M ammonium acetate in 80% v/v methanol was added to the phenolic phase collected and left overnight at −20°C. The sample was again centrifuged (12,300 *g*, 10 min, 4°C) to obtain the protein pellet that was then rinsed first with methanol and next with 80% v/v acetone, twice. The protein pellet was briefly air-dried and resuspended in 200 µL Laemmli buffer.

Protein concentration was determined by Coomassie blue dye-binding method using BSA as a standard (Bradford, 1976).

Thereafter, protein samples were diluted with MilliQ water and LDS3X reagent (Invitrogen) to obtain a 30 µL LDS1X solution containing 50 µg of proteins. After heating at 99°C for 5 min, proteins were loaded onto a NuPAGE 4−12% gradient gel (Invitrogen) and subjected to a short electrophoresis of 5 min. The samples were treated and proteolyzed with trypsin Gold (Promega) in presence of ProteaseMax detergent (Invitrogen) as previously described (Hartmann et al., 2014).

#### Liquid Chromatography and High Resolution Mass Spectrometry

NanoLC-MS/MS analysis of peptides was carried out in data-dependent mode with a Q-Exactive HF mass spectrometer (Thermo Fisher Scientific, USA) coupled to an UltiMate 3000 LC system (Dionex-LC Packings, Thermo Fisher Scientific). The analytical system was operated as described elsewhere (Klein et al., 2016). Peptides (10 µL) were desalted on a reverse-phase C18 PepMap 100 column, and then resolved with a 90 min gradient of CH_3_CN, 0.1% formic acid, at a flow rate of 0.2 µL min^-1^. The gradient was from 4 to 25% solvent B (80% CH_3_CN, 19.9% MilliQ water, 0.1% formic acid) against solvent A (99.9% MilliQ water, 0.1% formic acid) for 75 min and then from 25% to 40% for 15 min. Full scan mass spectra were acquired from *m/z* 350 to 1800 with an automatic gain control (AGC) target set at 3 × 10^6^ ions and a resolution of 60,000. The 20 most abundant precursor ions in each scan cycle were sequentially subjected to fragmentation through high-energy collisional dissociation. MS/MS scans were initiated for ions with potential charge states of 2^+^ and 3^+^ with an AGC target at 10^5^ ions and threshold intensity of 83,000. A dynamic exclusion of 10 sec was applied for improving peptide coverage.

#### Protein Identification and Label Free Quantification

MS/MS spectra were assigned to peptide sequences using the MASCOT Daemon 2.6.1 search algorithm (Matrix Science). A reference database from *C. canephora* (Denoeud et al., 2014) of 25,574 polypeptide sequences totaling 10,251,572 residues was downloaded from Genoscope (http://coffee-genome.org/sites/coffee-genome.org/files/download/coffea_cds.fna.gz) on July 1^st^ 2019 and used for peptide and protein inference. The following parameters were used in the search: trypsin as proteolytic enzyme, maximum of two missed cleavages, mass tolerances of 5 ppm on the precursor ion and 0.02 Da on the MS/MS, fixed modification of cysteine into carboxyamidomethylated cysteine (+57.0215), and oxidized methionine (+15.9949) as variable modification. All peptide matches with a MASCOT peptide score below a *p* value of 0.05 were filtered and assigned to a protein. In order to keep the biologically relevant protein isoforms typical from plant proteomes, parsimony was not applied between samples. A protein was validated when at least two different peptide sequences were detected. The false discovery rate for protein identification was estimated through the decoy search option of MASCOT (Matrix Science) to be below 1%. Label-free quantification was based on counts of peptide-to-spectrum matches for each polypeptide.

From our results we searched for proteins that are known to be involved in cyclic electron transport, and four proteins were found: one proton gradient regulation protein PGR5 (*Cc08_g13730 - PGR5-like protein 1A, chloroplastic*) and three chloroplast NADH dehydrogenase-like (NDH) complex proteins (*Cc06_g22880 - Putative NDH-dependent cyclic electron flow 5, Cc04_g05100 - NDH-dependent cyclic electron flow 1, Cc06_g22890 - NDH-dependent cyclic electron flow 1*). The mass spectrometry proteomics data have been deposited to the ProteomeXchange Consortium via the PRIDE partner repository with the dataset identifier PXD019474 and DOI: 10.6019/PXD019474 for *C. arabica* proteome, and the dataset identifier PXD019541 and DOI: 10.6019/PXD019541 for *C. canephora* proteome.

### Statistical Analysis

Physiological and biochemical data were analysed using a two-way ANOVA to evaluate the differences between water treatments (WW or SWD), between temperature treatments, and their interaction, followed by a Tukey’s test for mean comparisons.

For the proteomic data, a two-way ANOVA was used to evaluate the differences between water treatments, between the several temperatures, and their interaction, followed by a Fisher’s LSD test for mean comparisons between each condition and the double control (WW at 25/20°C).

A 95% confidence level was adopted for all tests, which were performed always independently for each genotype.

## Results

### Leaf Water Status

**Figure 1:**
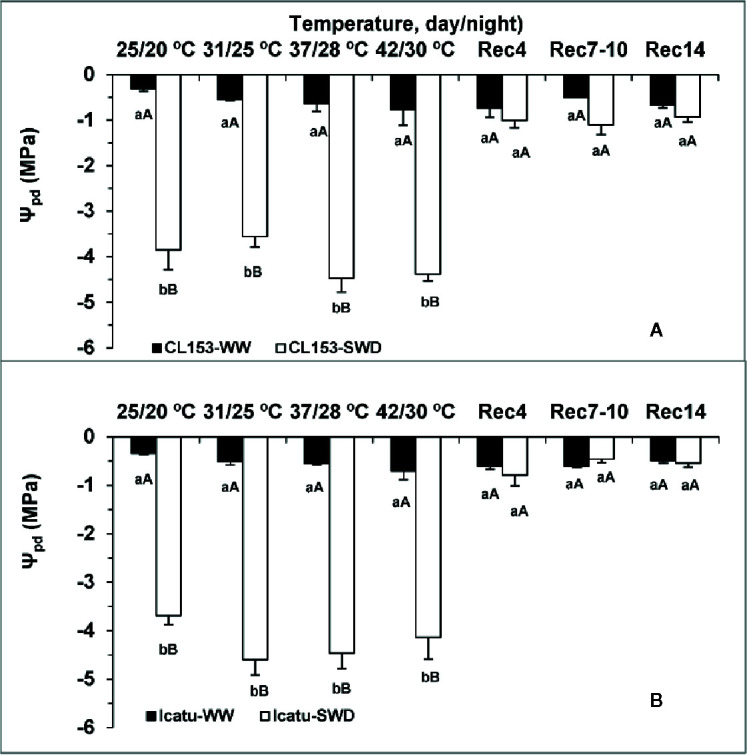
Leaf water potential (*Ψ_pd_*) determined at pre-dawn in *Coffea canephora* cv. Conilon Clone 153 (CL153) and *Coffea arabica* cv. Icatu, submitted to well-watered (WW) and severe drought (SWD), and temperature increase from (25/20°C, day/night), to 42/30°C, followed by a recovery of 4 (Rec4), 7 to 10 (Rec7-10) and 14 (Rec14) days. For each parameter, the mean values ± SE (n=5-6) followed by different letters express significant differences between temperature treatments for the same water level **(A, B)**, or between water availability levels for each temperature treatment (a, b), always separately for each genotype.

The *Ψ_pd_* was remarkably low (below −3.7 MPa) in SWD plants under control temperature (25/20°C), and tended to reach even lower values, close to −4.40 MPa at the two highest temperatures irrespective of genotype (**Figure 1**).

The single temperature rise did not significantly modify water status, despite the gradual decline tendency of *Ψ_pd_* in WW plants from 25/20°C to the highest temperature, *i.e.* from −0.31 to −0.77 MPa in CL153, and from −0.34 to −0.70 MPa in Icatu (**Figure 1**).

Along the recovery period, *Ψ_pd_* in SWD plants recovered from 4 days onwards to values close to their respective controls.

### Changes in Stomatal Traits Driven by Drought and/or Heat

**Figure 2:**
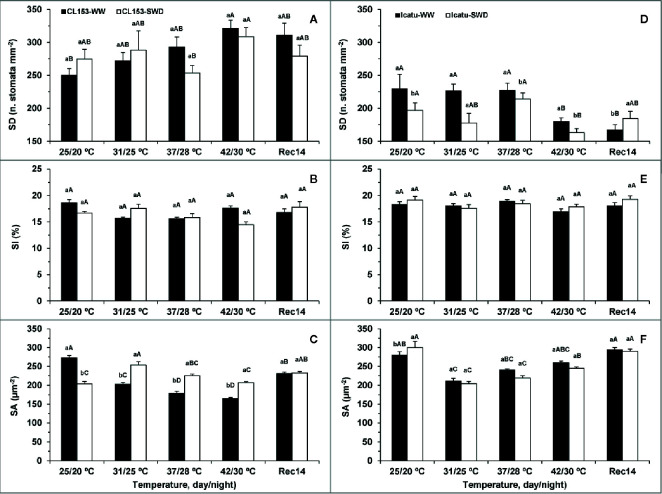
Stomatal density (SD) **(A, D)**, stomatal index (SI) **(B, E)** and stomatal area (SA) **(C, F)** from leaves of *Coffea canephora* cv. Conilon Clone 153 (CL153) and *Coffea arabica* cv. Icatu, submitted to well-watered (WW) and severe drought (SWD), and temperature increase from (25/20°C, day/night), to 42/30°C, followed by a recovery of 14 days (Rec14) days. For each parameter, the mean values ± SE (n=5) followed by different letters express significant differences between temperature treatments for the same water level **(A–D)**, or between water availability levels for each temperature treatment (a, b), always separately for each genotype.

The stomatal density (SD) was not significantly altered by drought in CL153, but it gradually increased with the imposition of heat, although significantly only at 42/30°C (**Figure 2**). In contrast, SD was reduced in Icatu with the single imposition of either severe drought or heat stress, reaching the lowest values upon stress superimposition (SWD plants at 42/30°C). Two weeks after stress relief the SD values remained similar to those at 42/30°C.

The single exposure to drought (at 25/20°C) reduced the stomata area (SA) in CL153, with an opposite behavior in Icatu, whereas elevated temperature alone (in WW plants) reduced SA in both genotypes from 31/25°C up to 42/30°C (**Figure 2**). The exposure of SWD plants to all supra-optimal temperatures attenuated SA decline in CL153 with no clear impact in Icatu since SA was similar regardless of water treatments, although lower than at 25/20°C.

At the end of the experiment (Rec14), WW and SWD plants showed similar values of SD and SA within each genotype, but a fully recovery to the initial WW values were observed only in Icatu for SA and CL153 for SD.

The stomatal index (SI) was unresponsive to the applied treatments.

### Stresses Impact on Leaf Gas Exchanges

Compared to WW plants, single exposure to drought significantly depressed the net photosynthetic rate (*P_n_*) to 18% and 8% in CL153 and Icatu plants, respectively (**Table 1**). Concomitant reductions of stomatal conductance (*g_s_*) (to 25 and 23%), and transpiration rates (*E*) (to 33 and 40%) were observed, in the same genotype order. Instantaneous water use efficiency (*WUE*) was also reduced due to a stronger decrease in *P_n_* than in *E*. In contrast, the internal [CO_2_] (*C_i_*) showed an almost doubled value in SWD than in WW plants at 25/20°C. Reductions in photosynthetic capacity (*A_max_*) of SWD plants, to 59% (CL153) and 79% (Icatu), were found relative to their respective WW values.

**Table 1:** Variation in the leaf gas exchange parameters, net photosynthesis (*P_n_*), stomatal conductance to water vapor (*g_s_*), transpiration (*E*), and photosynthetic capacity (*A_max_*) rates, as well as values of internal concentration of CO_2_ (*C_i_*), instantaneous water-use efficiency (*WUE*) in *Coffea canephora* cv. Conilon Clone 153 (CL153) and *Coffea arabica* cv. Icatu, submitted to well-watered (WW) and severe drought (SWD), and temperature increase from (25/20°C, day/night), to 42/30°C, followed by a recovery of 4 (Rec4), 7-10 (Rec7-10) and 14 (Rec14) days.

Genotype	Water	Temperature (day/night)									
		25/20°C	28/23°C	31/25°C	34/28°C	37/28°C	39/30°C	42/30°C	Rec4	Rec7-10	Rec14
		***P_n_* (µmol CO_2_ m^-2^ s^-1^)**									
**CL153**	**WW**	5.68 ± 0.48aA	3.85 ± 0.46aAB	3.72 ± 0.25aAB	3.29 ± 0.51aBC	4.20 ± 1.17aAB	1.23 ± 0.18aC	1.24 ± 0.30aC	2.91 ± 0.35aBC	3.11 ± 0.20aBC	4.33 ± 0.10aAB
	**SWD**	1.01 ± 0.65bA	0.43 ± 0.19bB	−0.09 ± 0.16bB	0.00 ± 0.25bB	−0.16 ± 0.25bB	−0.25 ± 0.07bB	−0.48 ± 0.18bB	1.51 ± 0.29bB	3.87 ± 0.59aA	4.13 ± 0.54aA
**Icatu**	**WW**	5.06 ± 0.40aAB	4.43 ± 0.41aABC	4.16 ± 0.37aABCD	2.77 ± 0.26aCD	2.76 ± 0.36aCD	2.71 ± 0.39aD	2.85 ± 0.41aCD	4.17 ± 0.15aBCD	5.31 ± 0.44aAB	6.12 ± 0.48aA
	**SWD**	0.41 ± 0.29bCD	0.14 ± 0.26bD	−0.35 ± 0.07bD	−0.01 ± 0.26bD	0.15 ± 0.16bD	−0.13 ± 0.07bD	−0.13 ± 0.15bD	1.87 ± 0.35bC	3.61 ± 0.65bB	5.39 ± 0.46aA
		***g_s_* (mmol H_2_O m^−2^ s^−1^)**									
**CL153**	**WW**	43.3 ± 6.9aAB	52.8 ± 2.6aA	32.7 ± 4.2aAB	32.8 ± 3.2aAB	52.6 ± 11.2aA	27.7 ± 8.5aB	21.3 ± 4.2aB	27.7 ± 3.6aB	36.6 ± 3.6aAB	37.2 ± 3.5aAB
	**SWD**	10.7 ± 6.8bC	9.7 ± 2.2bC	10.6 ± 1.7bC	7.4 ± 1.1bC	9.0 ± 1.5bC	5.6 ± 0.6bC	7.0 ± 1.0bC	17.0 ± 3.5aBC	50.9 ± 8.3aA	40.0 ± 8.7aAB
**Icatu**	**WW**	48.7 ± 5.1aBC	43.5 ± 8.8aC	42.7 ± 8.8aC	29.3 ± 2.9aC	28.6 ± 4.6aC	51.3 ± 8.6aBC	77.4 ± 12.8aAB	57.8 ± 3.6aABC	78.5 ± 12.3aAB	77.7 ± 13.2aAB
	**SWD**	11.4 ± 2.7bC	6.6 ± 1.0bC	6.2 ± 0.8bC	6.6 ± 1.4bC	4.8 ± 1.2bC	6.3 ± 0.4bC	5.0 ± 0.5bC	19.4 ± 1.8bBC	49.0 ± 6.7bAB	66.8 ± 9.5aA
		***Ci* (µL L^−1^)**									
**CL153**	**WW**	160.2 ± 29.1bB	248.3 ± 20.9bA	166.5 ± 18.3bB	250.2 ± 15.0bA	235.0 ± 19.0bAB	257.5 ± 17.0bA	267.2 ± 16.3bA	186.0 ± 5.8aAB	218.7 ± 7.5aAB	155.0 ± 15.4aB
	**SWD**	312.0 ± 62.9aABCD	352.2 ± 37.9aABC	397.4 ± 23.7aA	341.6 ± 51.5aABC	376.7 ± 42.5aAB	410.4 ± 13.9aA	422.8 ± 30.5aA	218.2 ± 36.3aCD	251.7 ± 11.0 aBCD	184.3 ± 27.2aD
**Icatu**	**WW**	166.7 ± 44.1bA	203.0 ± 67.4bA	197.9 ± 29.8bA	220.1 ± 20.3bA	194.7 ± 27.0bA	258.3 ± 5.3bA	267.8 ± 15.3bA	237.2 ± 3.2aA	233.7 ± 12.3aA	204.8 ± 14.7aA
	**SWD**	317.5 ± 20.6aBCDE	348.4 ± 53.0aABCD	452.4 ± 15.8aA	345.2 ± 47.5aA	274.8 ± 32.2aABCD	378.6 ± 17.7aABC	414.7 ± 21.5aAB	213.5 ± 20.8aE	205.3 ± 17.8aE	253.0 ± 41.6aDE
		***E* (mmol H_2_O m^−2^ s^−1^)**									
**CL153**	**WW**	0.409 ± 0.066aD	0.835 ± 0.104aBC	0.951 ± 0.053aBCD	1.926 ± 0.462aA	1.818 ± 0.371aAB	1.328 ± 0.287aABC	1.201 ± 0.210aABCD	0.520 ± 0.055aCD	0.567 ± 0.049aCD	0.616 ± 0.064aCD
	**SWD**	0.136 ± 0.086aA	0.209 ± 0.037bA	0.406 ± 0.065bA	0.310 ± 0.047bA	0.480 ± 0.034bA	0.329 ± 0.033bA	0.479 ± 0.071bA	0.354 ± 0.070aA	0.640 ± 0.076aA	0.549 ± 0.093aA
**Icatu**	**WW**	0.362 ± 0.064aD	0.923 ± 0.190 aCD	0.916 ± 0.081aCD	1.134 ± 0.069aC	1.278 ± 0.217aC	2.080 ± 0.237aB	2.938 ± 0.435aA	1.015 ± 0.071aCD	1.003 ± 0.050aCD	0.969 ± 0.080aCD
	**SWD**	0.145 ± 0.023aB	0.089 ± 0.050bB	0.208 ± 0.034bAB	0.280 ± 0.056bAB	0.245 ± 0.056bAB	0.362 ± 0.022bAB	0.327 ± 0.040bAB	0.401 ± 0.030bAB	0.540 ± 0.084bAB	0.833 ± 0.116aA
		***WUE* (mmol CO_2_ mol^−1^ H_2_O)**									
**CL153**	**WW**	12.10 ± 2.66aA	3.71 ± 0.29aCD	3.98 ± 0.22aBCD	2.18 ± 0.36aCD	2.63 ± 0.57aCD	1.03 ± 0.16aD	0.87 ± 0.19aD	5.54 ± 0.11aBC	5.60 ± 0.28aBC	8.04 ± 1.01aAB
	**SWD**	4.58 ± 1.74bBC	2.79 ± 1.17aBCD	0.74 ± 0.33bCD	2.80 ± 1.18aBCD	2.23 ± 1.62aBCD	−0.72 ± 0.15aD	0.61 ± 0.05aCD	4.74 ± 1.00aBC	5.85 ± 0.61aAB	9.10 ± 1.80aA
**Icatu**	**WW**	15.31 ± 3.19aA	4.53 ± 0.91aB	4.38 ± 0.78aB	2.16 ± 0.23aB	3.05 ± 0.87aB	1.29 ± 0.10aB	1.18 ± 0.19aB	4.18 ± 0.14aB	5.27 ± 0.30aB	6.38 ± 0.22aB
	**SWD**	3.03 ± 0.95bAB	2.39 ± 1.16aAB	3.42 ± 2.29aAB	3.85 ± 2.06aAB	1.18 ± 0.46aB	0.18 ± 0.09aB	0.13 ± 0.02aB	4.50 ± 0.62aAB	6.85 ± 0.83aA	7.25 ± 0.82aA
		***A_max_* (µmol O_2_ m^−2^ s^−1^)**									
**CL153**	**WW**	12.1 ± 1.4aAB	–	13.8 ± 1.9aAB	–	13.5 ± 2.3aAB	–	8.3 ± 0.3aB	9.4 ± 1.8aAB	15.2 ± 2.2aA	12.8 ± 1.1aAB
	**SWD**	7.1 ± 0.7bAB	–	9.7 ± 2.4bAB	–	8.5 ± 1.1bAB	–	7.6 ± 2.5aAB	8.3 ± 0.9bAB	10.8 ± 0.8bA	10.3 ± 1.7bAB
**Icatu**	**WW**	11.1 ± 0.9aBC	–	15.3 ± 2.0aAB	–	10.8 ± 1.7aC	–	5.4 ± 1.0aD	13.5 ± 1.3aABC	16.4 ± 1.2aA	14.9 ± 1.0aABC
	**SWD**	8.8 ± 1.2bBC	–	12.0 ± 2.5bAB	–	7.8 ± 1.4bC	–	4.1 ± 0.4aD	10.8 ± 2.0bABC	13.0 ± 0.6bA	10.8 ± 1.8bABC

For each parameter, the mean values ± SE (n=6) followed by different letters express significant differences between temperature treatments for the same water level (A, B, C, D), or between water availability levels for each temperature treatment (a, b), always separately for each genotype.

The single exposure to high temperature (WW plants) caused different impacts between genotypes in these same parameters. *P_n_* was gradually reduced above 25/20°C, significantly only at 39/30 and 42/30°C in CL153, when the values represented only *ca*. 22% of those at 25/20°C. In contrast, Icatu was affected from 34/28°C onwards, but maintained *ca.* 55% of the initial *P_n_* values at these highest temperatures, more than doubling relative to CL153 values. This might have also contributed to an earlier recovery in Icatu from Rec4 onwards, and the partial recovery in CL153 by Rec14, when *P_n_* represented 76% of the control value.

The *A_max_* peaked at 31/25°C and decreased afterwards (especially in Icatu), although significantly only at 42/30°C, when it represented 69% and 49% of the values observed at 25/20°C in CL153 and Icatu, respectively.

Stomata opening responded differently to increasing temperatures relative to drought. In fact, *g_s_* suffered some fluctuations until 37/28°C in both genotypes, but above this temperature it was reduced in CL153 (accompanying *P_n_* trend), and increased in Icatu. This was in line with *E* values, which peaked at 37/28°C in CL153, whereas it increased continuously until 42/30°C in Icatu. At this temperature *E* values were 193% and 712% higher than those of controls of CL153 and Icatu, respectively. These increases in *E*, together with *P_n_* declines, led to remarkable *WUE* reductions. Noteworthy is also the fact that *C_i_* increased above 25/20°C, especially at the highest two temperatures, similarly for both genotypes, pointing to an absence of CO_2_ limitation to photosynthesis.

Along the recovery period Icatu kept *g_s_* and *E* values higher than those of control plants (and lower *WUE*), whereas CL153 maintained lowered *g_s_* (as *P_n_*) values until Rec 7-10, although with *E* and *C_i_* values closer to their control than did Icatu.

The combined stress exposure clearly aggravated most gas exchange impacts. For SWD plants of both genotypes *P_n_* further decreased to negligible (or negative) values from 28/23°C to 42/30°C (**Table 1**), whereas *g_s_* became residual. This strongly reduced the water loss by transpiration as compared to WW plants at each temperature, although *E* also increased in SWD plants accompanying the temperature rise. In any case,*WUE* values were usually similar to those of WW plants from 28/23 to 42/30°C in CL153 and Icatu plants.

For both genotypes, *A_max_* was lower in SWD than in WW plants until 37/28°C (although not differing from values at 25/20°C), suggesting that *A_max_* decreases were mostly imposed by drought than by heat until this temperature. However, at 42/30°C *A_max_* was further reduced in SWD Icatu plants, but without difference relative to WW plants, thus suggesting that at this extreme temperature heat played a major role for this additional decline of *A_max_*.

The greater severity imposed by stress combination was also reflected in the slower recovery of SWD plants, usually until Rec4, but by Rec14 close values of *P_n_*, *g_s_*, *C_i_*, *E* and *WUE* were observed between WW and SWD plants in both genotypes. Nevertheless, only Icatu showed a complete *P_n_* recovery in SWD plants, as compared to WW at the initial 25/20°C conditions (contrary to WW and SWD plants of CL153), while maintaining significantly higher *g_s_* and *E* values. Furthermore, only Icatu SWD plants showed a total recover of *A_max_* from Rec4 onwards, although keeping lower values than those of WW plants.

### Stress Promoted Alterations in Fluorescence Parameters

In both genotypes, drought (at 25/20°C) did not affect *F_0_*, whereas heat (in WW plants) led to significant increases only at 42/30°C (**Table 2**). Interestingly, at this temperature *F_0_* was less affected by the combined stress exposure, although a full recovery was faster in WW (Rec4) than in SWD plants (Rec7).

**Table 2:** Variation in the leaf chlorophyll *a* fluorescence parameters in *Coffea canephora* cv. Conilon Clone 153 (CL153) and *Coffea arabica* cv. Icatu, submitted to well-watered (WW) and severe drought (SWD), and temperature increase from (25/20°C, day/night), to 42/30°C, followed by a recovery of 4 (Rec4), 7-10 (Rec7-10) and 14 (Rec14) days.

Genotype	Water	Temperature (day/night)									
		25/20°C	28/23°C	31/25°C	34/28°C	37/28°C	39/30°C	42/30°C	Rec4	Rec7-10	Rec14
		***F_o_***									
**CL153**	**WW**	0.210 ± 0.004aB	0.216 ± 0.005aB	0.223 ± 0.007aB	0.195 ± 0.007aB	0.288 ± 0.021aB	0.211 ± 0.006aB	0.499 ± 0.038aA	0.265 ± 0.010bB	0.203 ± 0.011aB	0.248 ± 0.005aB
	**SWD**	0.233 ± 0.008aABC	0.275 ± 0.016aABC	0.204 ± 0.013aBC	0.182 ± 0.013aC	0.214 ± 0.018aBC	0.232 ± 0.017aABC	0.349 ± 0.020bA	0.353 ± 0.020aA	0.214 ± 0.008aBC	0.312 ± 0.007aAB
**Icatu**	**WW**	0.251 ± 0.007aB	0.282 ± 0.007aB	0.265 ± 0.006aB	0.224 ± 0.009aB	0.336 ± 0.016aB	0.214 ± 0.004aB	0.650 ± 0.062aA	0.270 ± 0.007aB	0.206 ± 0.017aB	0.237 ± 0.011aB
	**SWD**	0.244 ± 0.005aAB	0.241 ± 0.006aAB	0.220 ± 0.009aAB	0.217 ± 0.012aAB	0.226 ± 0.012bAB	0.246 ± 0.010aAB	0.343 ± 0.015bA	0.329 ± 0.009aAB	0.201 ± 0.003aB	0.295 ± 0.007aAB
		***F_v_/F_m_***									
**CL153**	**WW**	0.770 ± 0.008aAB	0.784 ± 0.005aA	0.794 ± 0.003aA	0.793 ± 0.006aA	0.789 ± 0.007aA	0.765 ± 0.008aAB	0.635 ± 0.025aC	0.728 ± 0.017aB	0.725 ± 0.013aB	0.759 ± 0.009aAB
	**SWD**	0.705 ± 0.025bAB	0.713 ± 0.026bAB	0.724 ± 0.024bAB	0.769 ± 0.008aA	0.759 ± 0.012aA	0.744 ± 0.015aAB	0.631 ± 0.021aC	0.673 ± 0.014bBC	0.688 ± 0.015bBC	0.718 ± 0.007bAB
**Icatu**	**WW**	0.753 ± 0.005aA	0.746 ± 0.006aA	0.766 ± 0.005aA	0.772 ± 0.009aA	0.744 ± 0.009aA	0.782 ± 0.005aA	0.458 ± 0.063bB	0.723 ± 0.011aA	0.736 ± 0.009aA	0.734 ± 0.010aA
	**SWD**	0.761 ± 0.008aA	0.778 ± 0.009aA	0.785 ± 0.008aA	0.766 ± 0.008aA	0.743 ± 0.004aAB	0.737 ± 0.014aAB	0.671 ± 0.011aB	0.704 ± 0.010aAB	0.718 ± 0.006aAB	0.719 ± 0.007aAB
		***Y_(II)_***									
**CL153**	**WW**	0.358 ± 0.044aABC	0.327 ± 0.033aABC	0.391 ± 0.027aAB	0.325 ± 0.036aABC	0.401 ± 0.013aA	0.279 ± 0.038aBC	0.219 ± 0.019aCD	0.138 ± 0.009aD	0.229 ± 0.051aCD	0.253 ± 0.028aBCD
	**SWD**	0.065 ± 0.010bB	0.194 ± 0.031bA	0.160 ± 0.030bAB	0.140 ± 0.021bAB	0.157 ± 0.021bAB	0.177 ± 0.036bAB	0.069 ± 0.008bB	0.190 ± 0.018aA	0.177 ± 0.022aAB	0.198 ± 0.012aA
**Icatu**	**WW**	0.356 ± 0.029aA	0.305 ± 0.012aABC	0.361 ± 0.014aA	0.301 ± 0.038aABC	0.309 ± 0.019aABC	0.318 ± 0.029aAB	0.085 ± 0.020aD	0.187 ± 0.013aCD	0.203 ± 0.013aBCD	0.265 ± 0.022aABC
	**SWD**	0.136 ± 0.013bABCD	0.227 ± 0.029aABC	0.206 ± 0.035bABC	0.120 ± 0.018bBCD	0.130 ± 0.019bABCD	0.096 ± 0.019bCD	0.065 ± 0.011aD	0.230 ± 0.027aAB	0.232 ± 0.019aAB	0.252 ± 0.020aA
		***Y_(NPQ)_***									
**CL153**	**WW**	0.245 ± 0.043bA	0.346 ± 0.033bA	0.245 ± 0.037bA	0.345 ± 0.024bA	0.243 ± 0.022bA	0.309 ± 0.056bA	0.476 ± 0.028bA	0.475 ± 0.032aA	0.238 ± 0.060aA	0.331 ± 0.036aA
	**SWD**	0.615 ± 0.021aAB	0.499 ± 0.033aAB	0.468 ± 0.050aABC	0.511 ± 0.027aAB	0.412 ± 0.047aABC	0.469 ± 0.072aABC	0.633 ± 0.022aA	0.376 ± 0.043aBC	0.225 ± 0.038aC	0.426 ± 0.028aABC
**Icatu**	**WW**	0.295 ± 0.034bABC	0.317 ± 0.021bAB	0.241 ± 0.015bBC	0.321 ± 0.034bAB	0.277 ± 0.017bABC	0.316 ± 0.035bAB	0.356 ± 0.039bAB	0.440 ± 0.021aA	0.163 ± 0.049aC	0.191 ± 0.024bC
	**SWD**	0.585 ± 0.026aAB	0.499 ± 0.034aABC	0.472 ± 0.061aBC	0.558 ± 0.047aABC	0.460 ± 0.045aBC	0.619 ± 0.039aAB	0.649 ± 0.021aA	0.410 ± 0.052aBC	0.114 ± 0.029aD	0.343 ± 0.028aC
		***Y_(NO)_***									
**CL153**	**WW**	0.397 ± 0.015aB	0.327 ± 0.018aB	0.364 ± 0.029aB	0.329 ± 0.016aB	0.356 ± 0.024aB	0.412 ± 0.032aB	0.305 ± 0.017aB	0.388 ± 0.034aB	0.533 ± 0.050aA	0.416 ± 0.025aB
	**SWD**	0.320 ± 0.026aB	0.306 ± 0.014aB	0.372 ± 0.030aB	0.349 ± 0.015aB	0.432 ± 0.035aB	0.354 ± 0.047aB	0.298 ± 0.019aB	0.434 ± 0.027aB	0.597 ± 0.034aA	0.376 ± 0.022aB
**Icatu**	**WW**	0.349 ± 0.018aC	0.378 ± 0.024aC	0.398 ± 0.014aBC	0.378 ± 0.026aC	0.414 ± 0.011aBC	0.366 ± 0.013aC	0.559 ± 0.049aAB	0.374 ± 0.017aC	0.634 ± 0.043aA	0.544 ± 0.026aAB
	**SWD**	0.280 ± 0.024aB	0.274 ± 0.014bB	0.322 ± 0.029aB	0.322 ± 0.035aB	0.410 ± 0.046aB	0.285 ± 0.028aB	0.287 ± 0.021bB	0.360 ± 0.028aB	0.654 ± 0.027aA	0.405 ± 0.021bB
		***q_L_***									
**CL153**	**WW**	0.448 ± 0.066aA	0.302 ± 0.039aABC	0.396 ± 0.030aAB	0.343 ± 0.029aABC	0.359 ± 0.023aABC	0.267 ± 0.055aABC	0.416 ± 0.052aAB	0.191 ± 0.044aC	0.235 ± 0.047aBC	0.237 ± 0.034aBC
	**SWD**	0.156 ± 0.030bA	0.241 ± 0.026aA	0.241 ± 0.042bA	0.208 ± 0.035bA	0.239 ± 0.032aA	0.309 ± 0.066aA	0.152 ± 0.020bA	0.314 ± 0.035aA	0.195 ± 0.021aA	0.234 ± 0.025aA
**Icatu**	**WW**	0.380 ± 0.029aA	0.332 ± 0.043aA	0.288 ± 0.027aA	0.316 ± 0.044aA	0.301 ± 0.033aA	0.371 ± 0.046aA	0.349 ± 0.107aA	0.184 ± 0.009aA	0.195 ± 0.013aA	0.223 ± 0.030aA
	**SWD**	0.205 ± 0.028bA	0.341 ± 0.042aA	0.286 ± 0.022aA	0.186 ± 0.024bA	0.193 ± 0.025aA	0.191 ± 0.045bA	0.151 ± 0.034bA	0.283 ± 0.033aA	0.240 ± 0.021aA	0.282 ± 0.022aA
		***F_v_’/F_m_’***									
**CL153**	**WW**	0.595 ± 0.023aAB	0.623 ± 0.036aAB	0.620 ± 0.024aAB	0.581 ± 0.025aAB	0.657 ± 0.015aA	0.614 ± 0.030aAB	0.413 ± 0.028aC	0.495 ± 0.043aBC	0.561 ± 0.038aABC	0.597 ± 0.024aAB
	**SWD**	0.355 ± 0.037bB	0.474 ± 0.035bAB	0.454 ± 0.039bAB	0.458 ± 0.018bAB	0.447 ± 0.035bAB	0.444 ± 0.056bAB	0.343 ± 0.026bB	0.441 ± 0.011bAB	0.515 ± 0.030bA	0.528 ± 0.025bA
**Icatu**	**WW**	0.593 ± 0.022aA	0.585 ± 0.028aA	0.671 ± 0.018aA	0.580 ± 0.031aA	0.608 ± 0.014aA	0.569 ± 0.015aA	0.283 ± 0.051aB	0.553 ± 0.014aA	0.567 ± 0.019aA	0.630 ± 0.018aA
	**SWD**	0.449 ± 0.020bABC	0.464 ± 0.025bABC	0.455 ± 0.039bABC	0.423 ± 0.035bABC	0.437 ± 0.024bABC	0.393 ± 0.028bBC	0.340 ± 0.023aC	0.508 ± 0.026aAB	0.559 ± 0.019aA	0.544 ± 0.019aA
		***F_s_/F_m_’***									
**CL153**	**WW**	0.642 ± 0.044bBC	0.673 ± 0.033bBC	0.609 ± 0.027bC	0.675 ± 0.036bBC	0.599 ± 0.013bC	0.721 ± 0.038bABC	0.781 ± 0.019bA	0.862 ± 0.009aA	0.771 ± 0.051aAB	0.747 ± 0.028aABC
	**SWD**	0.935 ± 0.010aA	0.806 ± 0.031aA	0.840 ± 0.030aA	0.860 ± 0.021aA	0.843 ± 0.021aA	0.823 ± 0.036aA	0.931 ± 0.008aA	0.810 ± 0.018aA	0.823 ± 0.022aA	0.802 ± 0.012aA
**Icatu**	**WW**	0.644 ± 0.029bC	0.695 ± 0.012aBC	0.639 ± 0.014bC	0.699 ± 0.038bBC	0.691 ± 0.019bBC	0.682 ± 0.029bBC	0.915 ± 0.020aA	0.813 ± 0.013aAB	0.797 ± 0.013aAB	0.735 ± 0.022aBC
	**SWD**	0.864 ± 0.013aABC	0.773 ± 0.029aBC	0.794 ± 0.035aBC	0.880 ± 0.018aABC	0.870 ± 0.019aABC	0.904 ± 0.019aAB	0.935 ± 0.011aA	0.770 ± 0.027aB	0.768 ± 0.019aB	0.748 ± 0.020aC

Parameters include the: initial fluorescence (F_o_), maximum PSII photochemical efficiency (F_v_/F_m_), photochemical quenching based on the concept of interconnected PSII antennae (q_L_), the actual PSII photochemical efficiency of energy conversion (F_v_’/F_m_’); and the predictor of the rate constant of PSII inactivation (F_s_/F_m_’), as well as the estimate of quantum yields of non-cyclic electron transport (Y_(II__)_), of regulated energy dissipation in PSII (Y_(NPQ__)_), and of non-regulated energy dissipation in PSII (heat and fluorescence) (Y_(NO)_).For each parameter, the mean values ± SE (n=5) followed by different letters express significant differences between temperature treatments for the same water level (A, B, C, D), or between water availability levels for each temperature treatment (a, b), always separately for each genotype.

The *F_v_/F_m_* was only significantly reduced by the single exposure to drought in CL153. Additionally, WW plants did not show impacts up to 39/30°C, but a large *F_v_/F_m_* reduction was observed at 42/30°C (mostly related to the *F_0_* rise) in both genotypes, although stronger in Icatu. No clear negative stress interaction was evident up to 42/30°C. Indeed, only a tendency to lower *F_v_/F_m_* values was observed at 39/30°C in SWD plants of both genotypes, and at 42/30°C the SWD Icatu plants showed even a 32% higher value than their WW counterparts. However, stress interaction was reflected along the recovery period given that SWD plants recovered more slowly and incompletely than WW ones in both genotypes.

The performance of the photosynthetic apparatus was further assessed under steady-state conditions. *Y_(II)_* was significantly reduced by single drought (larger in CL153) and 42/30°C (larger in Icatu). Notably, SWD plants tended to somewhat similar (Icatu) or higher (CL153) *Y_(II)_* values until 39/20°C, and even at 42/30°C drought seems to be the most important stress driver irrespective of genotype. Also, by Rec4 the SWD plants recovered slightly better than WW plants, but by the end of the experiment *Y_(II)_* did not completely recover regardless of genotypes and water conditions. Therefore, the *Y_(II)_* values until 42/30°C and in the recovery period did not clearly pointed to a stress interaction.

In both genotypes, *F_v_’/F_m_’* was significantly reduced by drought at 25/20°C (40% and 25%), and by 42/30°C (31% and 52%) in CL153 and Icatu, respectively, thus in line with *Y_(II)_* and *F_v_/F_m_* variations. Additionally, *q_L_* was only significantly reduced by drought (65% in CL153 and 46% in Icatu). However, it is noteworthy that under superimposed stress conditions *q_L_* values did not differ significantly between WW and SWD plants up to 39/30°C in CL153 and 31/25°C in Icatu. Also, at 42/30°C the SWD plants behaved similarly as their SWD counterparts at 25/20°C, again suggesting an absence of stress interaction and that drought was the most important limiting condition. Notably, *F_v_’/F_m_’* recovered completely in WW and SWD plants, faster in Icatu (Rec4) than in CL153 (Rec7-10). In turn, *q_L_* showed aftereffects by Rec14 regardless of treatments and genotypes.

A strong reinforcement of photoprotective energy dissipation mechanisms was reflected in *Y_(NPQ_*_)_ increases, under the single exposure of either stresses, especially under drought in both genotypes. Notably, the WW plants were not impacted until 39/30°C, but at 42/30°C clear rises (larger in CL153) of *Y_(NPQ_*_)_ were observed. With stress superimposition, SWD plants showed significantly higher values of these parameters than the WW plants at 42/30°C, but similar to those of SWD plants at 25/20°C, further pointing to an absence of stress interaction and that drought determined these genotype responses.

Notably, non-regulated energy dissipation processes (*Y_(NO_*_)_) were not significantly modified by drought or heat, except for WW Icatu plants at 42/30°C. Furthermore, under stress superimposition *Y_(NO_*_)_ tended to lower values in SWD plants than in WW ones at the two highest temperatures, pointing to an absence of aggravated status.

With some fluctuations, *Y_(NPQ)_* recovered mostly by Rec7-10, although the SWD plants showed higher values than those of WW ones by the end of the experiment, suggesting that some reinforcement of energy dissipation mechanisms are still needed in place. This was in line with some *Y_(NO_*_)_ rise by Rec7-10 (and in WW Icatu plants by Rec14).

PSII inactivation status (estimated as *F_s_/F_m_’*) nearly followed *F_v_’/F_m_’*, showing significant increases due to severe drought or heat (only at 42/30°C) conditions. In general, SWD plants from both genotypes maintained higher *F_s_/F_m_’* values than those of WW plants up to 42/30°C, although not differing from those at 25/20°C. These results also suggest an absence of stresses interaction.

Along the recovery period the *F_s_/F_m_’* values approached those of control, but with higher values even by Rec14. This was in line with the aftereffects in the energy driven to photochemical events (*Y_(II)_*) (all plants), and with some higher values of *Y_(NPQ)_* (SWD plants of both genotypes) or *Y_(NO_*_)_ (WW plants of Icatu).

### Stresses Impact in Thylakoid Functioning–Electron Transport Rates and Carriers

To unveil specific key impact points at the thylakoid membrane level, we next assessed the potential rates of electron transport involving PSs, and the content of the main carriers involved in electron transport.

**Figure 3:**
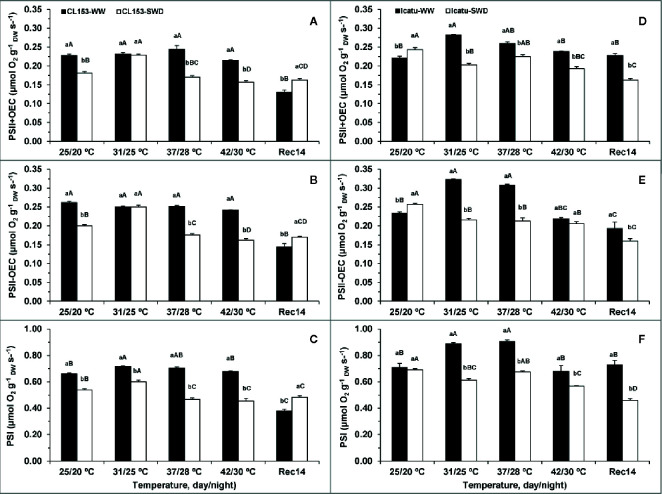
Changes in the potential thylakoid electron transport rates associated with *PSI* **(C, F)**, and *PSII*, with (+OEC) **(A, D)** or without (-OEC) **(B, E)** the oxygen evolving complex participation, in *Coffea canephora* cv. Conilon (CL153) and *Coffea arabica* cv. Icatu, submitted to well-watered (WW) and severe drought (SWD), and temperature increase from (25/20°C, day/night), to 42/30°C, followed by a recovery of 14 days (Rec14) days. For each parameter, the mean values ± SE (n=3) followed by different letters express significant differences between temperature treatments for the same water level **(A–D)**, or between water availability levels for each temperature treatment (a, b), always separately for each genotype.

In CL153, drought moderately reduced the activities of PSII including (PSII+OEC) or excluding (PSII-OEC) OEC, and of PSI in *ca.* 21, 24, and 18%, respectively (**Figure 3**), thus to a much smaller extent than in *Y_(II)_* (**Table 2**) or even *A_max_* (**Table 1**). Remarkably, SWD Icatu plants showed significant increases of *ca.* 10% in PSII activity (with or without OEC), whereas that of PSI remained unaffected.

The single exposure to heat did not impair PSs activities until 37/28°C for both genotypes. Furthermore, at the highest temperature no impact at the PSI level was observed in both genotypes, whereas PSII activity was marginally reduced only in CL153, as compared to the WW plants at 25/20°C. Yet, a closer look revealed that CL153 displayed maximum PSII and PSI activities at 37/28°C and 31/25°C, whereas in Icatu this happened at 31/25°C and 37/28°C, respectively. Taking this into account, both genotypes revealed some heat impact at 42/30°C when compared to their respective maxima. These impacts were stronger in Icatu which showed reductions of 16, 32, and 25%, for PSII+OEC, PSII-OEC, and PSI, respectively, against 12, 4, and 5% in CL153, in the same order.

Stress interaction exacerbated the impact on both PSs regardless of genotype, usually at 37/28°C and 42/30°C, when SWD plants presented lower PSs activities relative to (i) WW plants at these temperatures and (ii) SWD plants at 25/20°C. These reductions in SWD plants at 42/30°C reached *ca*. 37% for both PSII and PSI as compared to their maxima, but to a much lower extent than in WW plants at 25/20°C, and still held relevant activity under these very harsh conditions.

Unexpectedly, some of the strongest impacts on electron transport rates at PSs level were found at the end of the recovery period (Rec14). This was the case of WW CL153 plants that showed minimum rates, which were even below those of SWD plants in both PSs. Additionally, SWD plants showed strong aftereffects at the end of the experiment since the rates were maintained (CL153) or even reduced (Icatu) relative to those observed at maximal temperature. In contrast, WW Icatu displayed electron transport rates similar to those at the beginning of the experiment.

**Figure 4:**
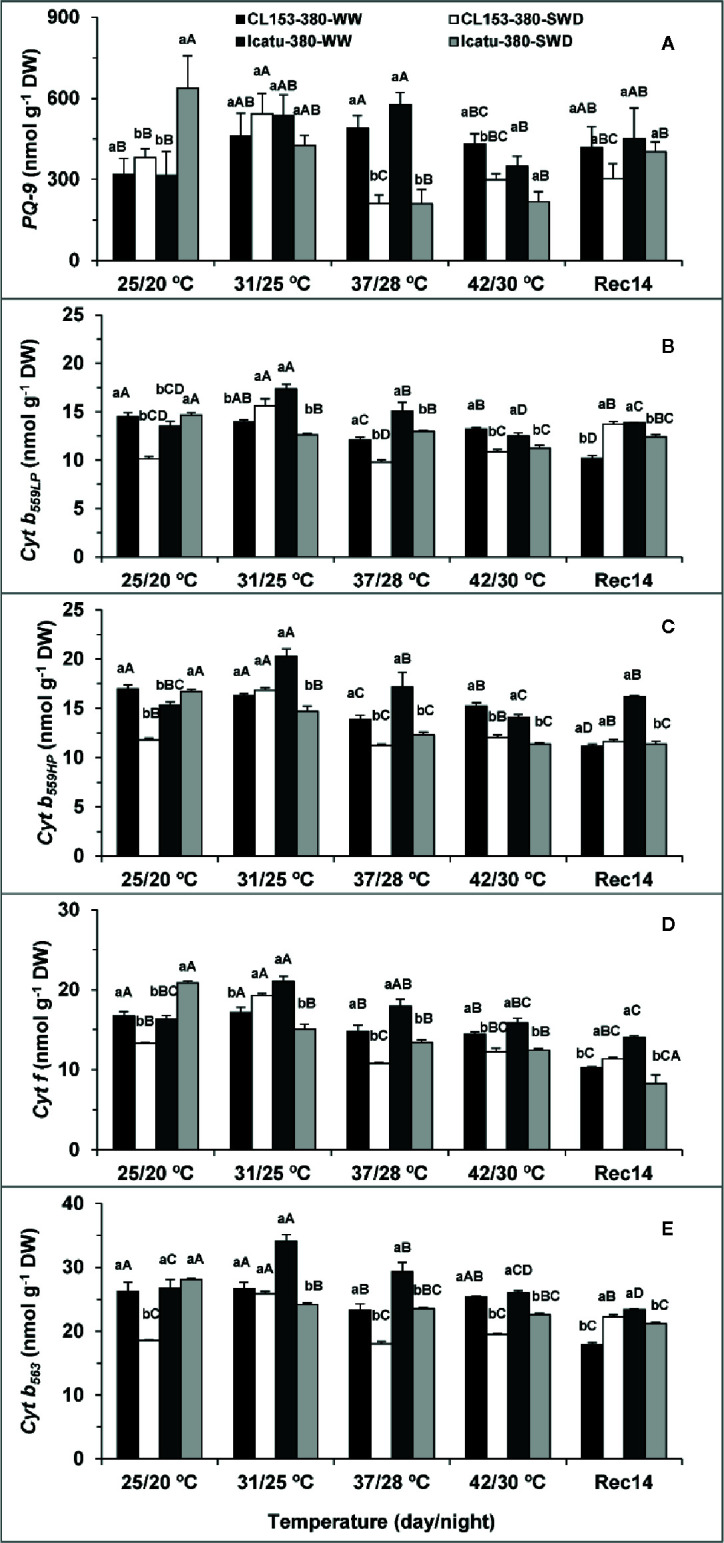
Variation in the contents of the thylakoid electron carriers plastoquinone (*PQ-9*) **(A)**, and cytochromes b*_559LP_* **(B)**, *b_559HP_* **(C)**; *b_563_* **(E)** and *f* **(D)** in *Coffea canephora* cv. Conilon (CL153) and *Coffea arabica* cv. Icatu, submitted to well-watered (WW) and severe drought (SWD), and temperature increase from (25/20°C, day/night), to 42/30°C, followed by a recovery of 14 days (Rec14) days. For each parameter, the mean values ± SE (n=3) followed by different letters express significant differences between temperature treatments for the same water level **(A–D)**, or between water availability levels for each temperature treatment (a, b), always separately for each genotype.

As concerns the contents of the thylakoid electron carriers (**Figure 4**), the single exposure to each stress promoted changes in a genotype-dependent manner, which, in most cases, closely followed the patterns of electron transport rates. Single drought increased the content of all cytochromes (Cyt) in Icatu, from 5% in *Cyt b_563_* (the only non-significant) to 28% in *Cyt f*, whereas *PQ-9* content doubled that of WW plants. In contrast, *PQ-9* was the only carrier to rise (20%) in CL153, whereas Cyt pools significantly declined between 20% (*Cyt f*) and *ca*. 30% (the rest).

At supra-optimal temperatures, the content of all electron carriers significantly increased at 31/25°C and 37/28°C in WW Icatu plants, ranging from *ca*. 30% (all Cyt at 31/25°C) to 83% in *PQ-9* (at 37/28°C). With a further increase to 42/30°C all contents were reduced, but maintaining values close to those at 25/20°C, similarly to what happened with electron transport rates (**Figure 3**). In contrast, the WW CL153 plants maintained Cyt contents at 31/25°C, but showed significant reductions between *ca*. 11–12% (*Cyt f*, *Cyt b_563_*) and 17–18% (*Cyt b_559HP_, Cytb_559LP_*) at 37/28°C. Despite a weak recovery at 42/30°C, significant differences to 25/20°C were still present (except in *Cyt b_563_*). The *PQ-9* was the only carrier to show large increases over the entire experiment (including 42/30°C), with a maximal 54% rise at 37/30°C.

The simultaneous stress exposure affected the carrier pools in both genotypes. The SWD plants showed lower contents of all carriers than their respective WW plants at 37/28°C and 42/30°C. Still, in CL153 the values were similar to those of SWD plants at 25/20°C, suggesting that the superimposition of stresses did not aggravate the drought impact that was already observed at 25/20°C. By opposition, the content of all electron carriers gradually decreased above 25/20°C in SWD Icatu plants, reaching minimum values at either 37/28°C or 42/30°C, thus pointing to an additional negative impact promoted by the stress interaction.

At Rec14, WW Icatu plants completely recovered (except *Cyt b_563_*) their carriers content, but in SWD plants lower values were still observed for most carriers (*Cyt b_559HP_*, *Cyt f*, *Cyt b_563_*), thus confirming the higher impact of stress superimposition. Notably, CL153 plants (WW and SWD) showed an incomplete recovery of all carrier pools (except for *PQ-9*) as compared to those found at 25/20°C. Furthermore, WW CL153 plants tended to lower values of all carriers than at 42/30°C, whereas the SWD plants recovered better than WW ones in some cases (*Cyt b_559LP_*, *Cyt b_563_*). These changes followed the same pattern observed for the electron transport rates (incomplete recovery of WW plants and higher values in SWD than WW plants at Rec14), thus further underlining the persistence of aftereffects by the end of the experiment.

### Proteins Involved in Cyclic Electron Flow

Under single drought exposure the abundance of PGR5 and NDH (*Cc04_g05100* and *Cc06_g22890*) proteins increased in SWD plants of both genotypes, although to a greater extent and significantly only in Icatu (**Table 3**).

**Table 3:** Changes in protein abundance (estimated by MS/MS spectra counts) of the proton gradient regulation protein PGR5 (*Cc08_g13730 - PGR5-like protein 1A, chloroplastic*) and three chloroplast NADH dehydrogenase-like (NDH) complex proteins (*Cc06_g22880 - Putative NDH-dependent cyclic electron flow 5, Cc04_g05100 - NDH-dependent cyclic electron flow 1, Cc06_g22890 - NDH-dependent cyclic electron flow 1*) in plants of *Coffea canephora* cv. Conilon Clone 153 (CL153) and *Coffea arabica* cv. Icatu, submitted to well-watered (WW) and severe drought (SWD), and temperature increase from (25/20°C, day/night), to 42/30°C, followed by a recovery of 14 days (Rec14) days.

Genotype	Water	Temperature (day/night)			
		25/20°C	37/28°C	42/30°C	Rec14
		***Cc06_g22880 - Putative NDH-dependent cyclic electron flow 5***			
**CL153**	**WW**	0.00	0.00	2.33	1.33
	**SWD**	0.00	0.33	1.00	0.00
**Icatu**	**WW**	0.00	0.00	0.00	0.00
	**SWD**	0.00	0.00	0.00	0.00
		***Cc04_g05100 - NDH-dependent cyclic electron flow 1***			
**CL153**	**WW**	0.33	1.00	2.33*	2.33*
	**SWD**	1.33	0.67	1.67	1.00
**Icatu**	**WW**	1.00	3.00*	2.67	2.33
	**SWD**	3.33*	1.67	1.67	4.00**
		***Cc06_g22890 - NDH-dependent cyclic electron flow 1***			
**CL153**	**WW**	2.00	3.00	3.33	4.67
	**SWD**	3.33	1.33	1.67	3.33
**Icatu**	**WW**	3.37	6.67	6.33	8.33*
	**SWD**	9.00**	6.00	4.00	6.67
		***Cc08_g13730 - PGR5-like protein 1A, chloroplastic***			
**CL153**	**WW**	0.00	3.67*	1.00	1.33
	**SWD**	0.33	1.00	0.67	2.00
**Icatu**	**WW**	3.33	7.67	1.00	4.67
	**SWD**	9.33*	1.67	0.33	3.67

Relative quantification was obtained by the number of MS/MS spectral counts associated with each protein. For each parameter, the mean values (n=3) followed by asterisks represented significant (*< 0.05) or very significant (**< 0.01) differences to the double control (WW plants, at 25/20°C), always separately for each genotype.

The levels of these three proteins increased by heat exposure in WW plants at 37/28°C, with Icatu showing again a greater responsiveness. However, at 42/30°C, PGR5 declined in both genotypes, whereas NDH (*Cc04_g05100* and *Cc06_g22890*) were mostly maintained in Icatu, and showed a rising tendency in CL153. At this temperature an additional NDH (*Cc06_g22880*) was also detected.

The superimposition of severe water deficit and heat compromised plant responses remarkably. In fact, at 37/28°C the proteins that increased under the single exposure to either stress (PGR5 and NDH, *Cc04_g05100* and *Cc06_g22890*) decreased in SWD plants, both as compared to their values at 25/20°C and to the values of WW plants at 37/28°C. At the highest temperature, abundance of these proteins was usually further reduced in SWD plants, and maintained at lower levels than in their WW counterparts irrespective of genotype.

Notably, two weeks after stresses relief PGR5 and NDH (*Cc04_g05100* and *Cc06_g22890*) protein pools were greater than their control initial values regardless of watering or genotypes.

### Impact on Key Photosynthetic Enzymes

**Figure 5:**
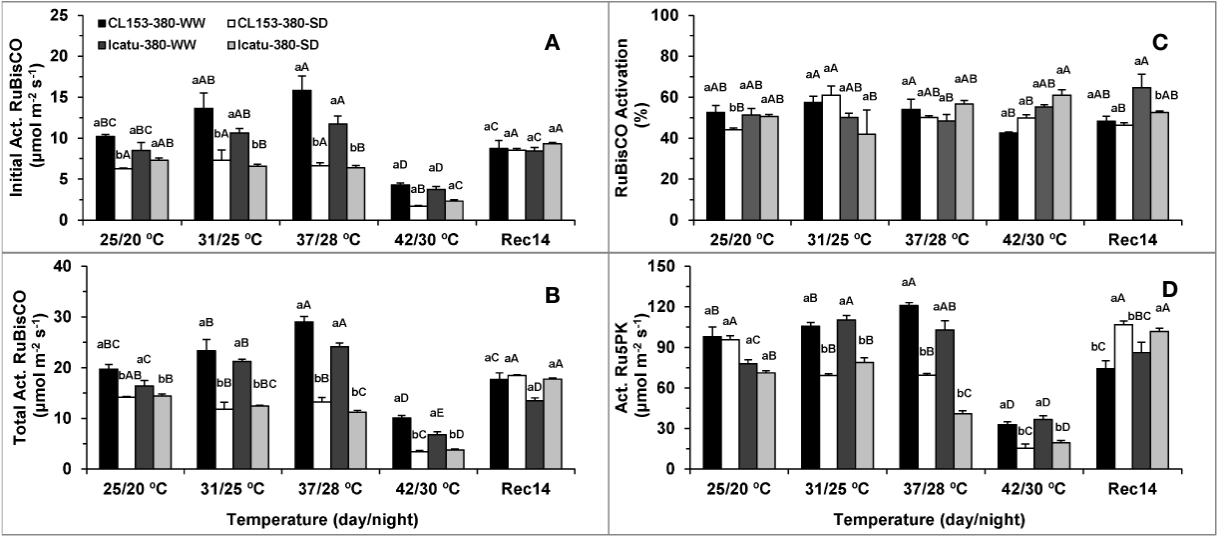
Changes in the Initial and Total Activities of ribulose-1,5-bisphosphate carboxylase oxygenase (RuBisCO), and this enzyme activation status, as well as the maximal activity of ribulose-5-phosphate kinase (Ru5PK), in plants of *Coffea canephora* cv. and *Coffea arabica* cv. Icatu, submitted to well-watered (WW) and severe drought (SWD), and temperature increase from (25/20°C, day/night), to 42/30°C, followed by a recovery of 14 days (Rec14) days. For each parameter, the mean values ± SE (n=5) followed by different letters express significant differences between temperature treatments for the same water level **(A–D)**, or between water availability levels for each temperature treatment (a, b), always separately for each genotype.

Drought significantly reduced RuBisCO initial (38%) and total (28%) activities, as well as its activation state (16%) in CL153 plants, whereas Icatu showed marginal reductions of 14, 12, and 1%, in the same order (**Figure 5**). Drought did not significantly affect Ru5PK activity in both genotypes.

The temperature rise up to 37/28°C significantly promoted RuBisCO and Ru5PK activities, while RuBisCO activation was maintained in WW plants of both genotypes. At this temperature, initial RuBisCO activity increased by 55% (CL153) and 38% (Icatu), and its total activity increased by 47% in both genotypes, whereas Ru5PK activity increased by 24% (CL153) or 32% (Icatu). However, these enzymes were particularly affected at 42/30°C irrespective of genotype, to a greater extent than was observed for the photochemical components. In fact, as compared to 25/20°C, the activities of RuBisCO (initial and total) and Ru5PK were nearly halved (or less) in WW plants from both genotypes. These decreases were even greater (*ca*. 65% or even more) when compared to the maximal activities at 37/28°C.

Notably, RuBisCO was mostly unresponsive to stress interaction until 37/28°C, whereas Ru5PK activity decreased significantly at this temperature in the SWD plants of both genotypes as compared to their counterparts at 25/20°C. However, at 42/30°C stress interaction clearly affected both enzymes and genotypes, with SWD plants displaying the lowest activities over the entire experiment. Under these harsh conditions (42/30°C and SWD), drastic reductions (*ca*. 80%) were observed in initial and total RuBisCO activities, as well as in Ru5PK activity in both genotypes, as compared to their controls. Such reductions were even greater if compared to WW plants at 37/28°C. Overall, RuBisCO activation remained unchanged, with a reduction only in WW CL153 plants of at 42/30°C.

At Rec14 enzyme activities recovered remarkably, although some aftereffects remained in RuBisCO (in WW and SWD plants of CL153 for initial activity; in WW Icatu plants for total activity) and Ru5PK activity (WW CL153 plants). Interestingly, both enzymes recovered completely in SWD Icatu plants, whereas in CL153 that was observed only for Ru5PK.

## Discussion

### Impact of Severe Drought on the Water Status and Photosynthetic Performance

Severe water restriction was imposed to SWD plants, as judged from the remarkable low *Ψ_pd_* (≤ −3.7 MPa) from 25/20°C to 42/30°C. This was below −3.5 MPa, which is considered an extreme water deficit in coffee trees (Pinheiro et al., 2004), or −2.15 MPa, which is low enough to cause leaf wilting (Santos and Mazzafera, 2012).

Water deficit and supra-optimal temperatures altered the stomatal traits SD and SA in a genotype-dependent manner, but not SI which is a reasonably constant trait in coffee (Grisi et al., 2008; Rodrigues et al., 2016). Under drought, Icatu showed reduced SD, similar to findings in droughted plants of *C. arabica* cv. Siriema (Melo et al., 2014), and a rise in SA. In contrast, CL153 presented opposite trends, denoting a different response to water constraints. Overall, decreased *g_s_* is associated with larger stomatal size and lower density for the same total area of stomatal pores, due to a larger diffusion path for water vapor (Franks and Beerling, 2009). Thus, SD and SA changes contributed to reduce *g_s_* in SWD Icatu plants. In CL153 such SD and SA changes would also at a first glance point to a *g_s_* increase.However the marked decreases in *g_s_* imply that the physiological regulation of stomata opening clearly overcomes the contribution of morphological stomatal traits over the control of water loss.

Coffee leaves display intrinsically low *g*_s_ values even under optimal growth conditions and thus, stomatal constraints, more than mesophyll or biochemical ones, have been shown to be the major limitations of photosynthesis (DaMatta et al., 2016; DaMatta et al., 2019; Martins et al., 2019). These constraints are believed to be exacerbated due to stomatal closure under soil drought (here shown by the strong *Ψ_pd_* decline) or rising leaf-to-air vapor pressure deficit, which in turn usually accompanies increases in air temperature (DaMatta et al., 2018). However, as drought severity progressed, non-stomatal factors dominate the limitations to photosynthesis, as herein shown by the increase in *C*_i_, despite the reduction in *g*_s_. This fact, coupled to reduction of *A_max_* (assessed under saturating CO_2_ and as such in the absence of diffusion-mediated limitations to photosynthesis) and to the greater decline of *P_n_* than in *A_max_*, clearly suggests that both biochemical and mesophyll constraints were the major factors explaining the overall photosynthesis decrease in SWD plants.

Single drought impact was further noted in the SWD plants in PSII photochemical efficiency (*F_v_/F_m_*, *F_v_’/F_m_’*) and inactivation (*F_s_/F_m_’*), the energy use to photosynthesis (*Y_(II)_*, *q_L_*), and the need for thermal energy dissipation (*Y_(NPQ)_*), with a global lesser impact in Icatu than in CL153, in agreement with the somewhat lower *A_max_* impact. Still, in both genotypes, the significant decline of *Y_(II)_* reflected a lower use of energy for ATP and NADPH synthesis (Peloso et al., 2017), in line with the very low *P_n_* values. Such lower photochemical use of energy was compensated for by the reinforcement of thermal dissipation mechanisms at the PSII level, reflected in strong *Y_(NPQ)_* rise, which protects the coffee leaves from excessive excitation damages (Pompelli et al., 2010; Silva et al., 2015). Besides, *Y_(NO)_* tended to lower values, meaning that photoinhibition and deregulated energy dissipation in PSII, related to limitations in photochemical processes (*Y_(II)_*) and/or insufficiency of photoprotective mechanisms (*Y_(NPQ)_*) (Kramer et al., 2004; Busch et al., 2009; Huang et al., 2011), did not occur in SWD plants.

Water deficit can cause protein denaturation (Hoekstra et al., 2001), decrease the synthesis of the small RuBisCO units, and increase RuBisCO inhibitors, thus affecting RuBisCO activity (Vu et al., 1999; Fahad et al., 2017). This is in good agreement with the greater drought sensitivity of CL153 plants, which showed stronger negative impacts on RuBisCO activity (and their activation state) (**Figure 5**) and both PSs activity (**Figure 3**), as well as in Cyt contents (**Figure 4**) than did Icatu. In fact, the higher photochemical performance in SWD Icatu plants was likely related to the preservation or reinforcement of those photosynthetic components associated with a strengthened antioxidative system under drought (Ramalho et al., 2018b). Still, PQ-9 increased in both genotypes, especially in Icatu, likely reinforcing the protective mechanisms against drought. In fact, PQ-9 corresponds to the redox form of plastoquinone (PQ) that displays antioxidant properties, capable of suppressing singlet oxygen (^1^O_2_) and inhibiting the oxidation of lipid membranes (Ksas et al., 2018). Furthermore, PQ is also linked to alternative electron flow pathways, among them the CEF involving PSII (with Cyt *b_559_*) and PSI (with Cyt *b_6_*/*f* complex). This is in good agreement with the increase in all Cyts displayed by SWD Icatu plants, in sharp contrast with the reductions found in CL153 plants. In fact, CEF can help to dissipate the excess photon energy and to mitigate PSs photoinhibition (Miyake and Okamura, 2003; Chu and Chiu, 2016; Yamori et al., 2016). For instance, Cyt *b_559_* (both LP and HP forms) is not involved in the primary electron transfer pathway in PSII, but can participate in CEF-PSII. This is not accompanied by O_2_ evolution, but significantly reduce the excess excitation pressure on PSII (Laisk et al., 2006), thus protecting it against photoinhibition (Shinopoulos and Brudvig, 2012; Chu and Chiu, 2016). Furthermore, the reinforcement of Cyt *b_563_* and *f* in SWD Icatu plants might have additionally promoted a CEF-PSI without accumulation of NADPH, but allowing the transport of H^+^ into the thylakoid lumen associated with the Q cycle, thus ultimately contributing to ΔpH formation and ATP synthesis. CEF-PSI further involves proton gradient regulation proteins (PGR5 and PGRL1), which mediate electron transport from ferredoxin to PQ, depending uniquely on the Q cycle of the Cyt *b_6_*/*f* complex. Additionally, a second CEF-PSI is related to the chloroplast NADH dehydrogenase-like (NDH) complex, which recycles electrons from ferredoxin to PQ and subsequently to PSI, alleviating oxidative pressure in chloroplasts under excessive light energy. Both PGR- and NDH dependent CEF-PSI were reported as essential for photoprotection of PSs under high irradiance and heat (Yamori et al., 2016; Shikanai and Yamamoto, 2017; Sun et al., 2018). Furthermore, the Cyt *b_6_*/*f* complex is a key control point of photosynthetic flow, and changes in electron transport capacity and C-assimilation are closely related to their content, which is sensitive to changes in environmental disturbances, including drought (Kohzuma et al., 2009; Sanda et al., 2011; Schöttler and Toth, 2014). Altogether, the greater increases in PGR5 and two NDH proteins as well as in all electron carriers observed in Icatu agree with CEF-PSII and CEF-PSI reinforcement, maintenance of PSII photochemical efficiency (*F_v_/F_m_*) and both PSs activity in SWD Icatu plants. In contrast, CL153 plants showed significant declines in *F_v_/F_m_*, PSs activity, reductions in all Cyt contents in addition to minor changes in PGR5 and NDH proteins. Similar impacts at PSI and PSII levels were associated with drought sensitivity in other plant species (Oukarroum et al., 2009; Chen et al., 2016), and showed that Icatu displayed a greater drought tolerance than CL153.

### Impact of Increasing Temperatures at Physiological and Biochemical Levels

Heat impact on photosynthesis was unrelated to leaf dehydration given that *Ψ_pd_* (**Figure 1**) was not affected by temperature rise despite the strong *E* increase (**Table 1**). Therefore, the potential impacts on photosynthetic performance at the imposed temperatures should have been mostly associated with metabolic disturbances or structural damages rather than changed leaf water status.

Temperature rise altered stomatal traits differently from drought, but also in a genotype-dependent manner. Although the SD increase and SA reduction observed in CL153 might have the potential to facilitate leaf cooling through a higher *g_s_*, and in Icatu the SD decrease could have promoted a decreasing *g_s_* trend at 42/30°C, none of these *g_s_* patterns were observed, *i.e.* CL153 showed the lowest, and Icatu the highest, *g_s_* values at 42/30°C. Therefore, as for drought, these findings highlights a greater importance of stomatal opening control, which overrode the contribution of altered stomatal traits in WW plants, confirming previous reports (Rodrigues et al., 2016).

CL153 showed a strong reduction of *P_n_* (and *g_s_*) at 39/30°C onwards, coupled with significant *C_i_* rise and *A*_max_ reduction, although with a much smaller extent than in *P_n_*. Together this suggests both stomatal and non-stomatal limitations of photosynthesis. In Icatu, the global photosynthetic functioning was even more affected at the highest temperature. Since higher *g_s_* and *C_i_* values were observed, concomitantly with a halved *A_max_*, we contend that non-stomatal limitations should have dominated the overall *P_n_* decreases. The dichotomous *P_n_* and *E* patterns contributed thereafter for the reduction of *WUE* to very low values in both genotypes, as also observed for whole coffee plants subjected to elevated temperatures (Rodrigues et al., 2018).

With the gradual temperature imposition Icatu presented maximal values of PSII activity and Cyt contents at 31/25°C (and for PSI activity and PQ-9 content at 37/28°C), supporting the significant *A_max_* rise. For both genotypes, a greater heat tolerance than that observed for severe water restriction was still found until 39/30°C in WW plants. This was reflected in the absence of significant changes in the energy capture in the antennae (*F_0_*), PSII photochemical efficiency (*F_v_/F_m_*, *F_v_’/F_m_’*) and inactivation (*F_s_/F_m_’*), photochemical energy use (*Y_(II)_*, *q_L_*), or even the energy dissipation mechanisms (*Y_(NPQ)_*, *Y_(NO)_*). Indeed, maintenance of high values of *Y_(II)_* and PSII photochemical efficiency reflect photosynthetic tolerance to stress (Li et al., 2017). This is likely to have been coupled with larger pools of several protective and antioxidative molecules, and the upregulated expression of some genes related to protection mechanisms, as previously demonstrated (Martins et al., 2016). Besides, photochemical quenching (represented by *q_L_*) is considered an indicator of PSII redox state as well as of energy captured by open PSII centers and used for electron transport (Murchie and Lawson, 2013). The higher the value, the greater the number of open reaction centers, reflecting a greater use of light by the plant, as happened here until 42/30°C in both genotypes.

Notably, at 31/25°C and even at 37/28°C a global rise of all Cyts (only in Icatu) and PQ-9 (both genotypes) was noted. At 37/28°C this was accompanied by higher values of PGR5 and two NDH (*Cc04_g05100* and *Cc06_g22890*), always greater in Icatu, whereas RuBisCO and Ru5PK showed maximal activities (both genotypes). This suggests a global investment in photosynthetic/chloroplast structures, in line with the strong lipid synthesis observed until 37/30°C, particularly in Icatu (Scotti-Campos et al., 2019). Such reinforcement of electron carriers, PGR5 and two NDH proteins, further highlights the presence of photoprotective CEF at PSI (Yamori et al., 2016; Sun et al., 2018) and PSII (Miyake and Okamura, 2003; Chu and Chiu, 2016) levels until 37/28°C, similarly to what was observed for drought conditions in Icatu. This complemented the antioxidative defences (*e.g*., enzymes such as SOD, and APX, Martins et al., 2016), while maintaining ATP synthesis that can be used, among others, for *de novo* protein synthesis needed for the rapid repair of photodamaged PSII (Murata et al., 2007; Huang et al., 2018). Altogether, these responses would support the maintenance of high PSs performance (Rodrigues et al., 2016) or even an upregulation of the photosynthetic apparatus at 31/25°C (with maximal Cyt and *A_max_* values in Icatu) and up to 37/28°C, despite the lower *P_n_* values.

Membrane stability is a crucial feature to drought and heat tolerance (Elbasyoni et al., 2017). Furthermore, thylakoid membranes are considered highly sensitive to heat, and impacts on photochemistry are among the first indicators of sensitivity, with damages occurring at PSII and chloroplast ultrastructure (Mano, 2002). This seemed to occur only at 42/30°C, when most fluorescence parameters were significantly altered in WW plants, usually to a higher extent in Icatu, namely in PSII photochemical efficiency. *F_0_* rise (accompanied by *F_v_/F_m_* decline) may reflected the uncoupling of LHCII from the PSII reaction center (Ruban, 2016). This rise indicates that a threshold for irreversible photoinhibition on the PSII centers have been exceeded (Pastenes and Horton, 1999; Baker and Rosenqvist, 2004), and it has been used to estimate crop tolerance to high temperature. Furthermore, *F_0_* rise might have been related to an over fluidity of chloroplast membranes (Tovuu et al., 2013) associated with altered membrane properties and loss of fatty acids from 37/28°C to 42/30°C, as previously observed in these genotypes (Scotti-Campos et al., 2019), These impairments found in both genotypes at 42/30°C, are in line with the stronger increase in PSII inactivation (*F_s_/F_m_’*), the *A_max_* decline, and the increase of non-regulated energy dissipation processes (*Y_(NO)_*), especially in Icatu (**Tables 1** and **2**). Such *Y_(NO)_* increase is usually associated with constraints in the use of incident radiation (Huang et al., 2011), which in turn is largely related to an inability for photochemical energy conversion (reduced *Y_(II)_*) as found in WW Icatu plants. Finally, this agrees with the rise in the rate constant for PSII inactivation (*F_s_/F_m_’*) as well as with the lowest values of *F_v_/F_m_* and *F_v_’/F_m_’* in WW Icatu plants. Overall, these results suggest a higher sensitivity of Icatu than CL153 only under extreme heat. However, PSII activity maintained most of its potential (**Figure 2**), whereas PSs activities (**Figure 3**) and electron carrier contents (**Figure 4**) suffered only minor impacts at the maximal temperature in both genotypes, as compared to their respective controls. This contrasts with reports of heat sensitivity of PSII (namely at D1 protein and OEC level) (Komayama et al., 2007) and PSI (Ivanov et al., 2017; Chovancek et al., 2019) in other species, likely associated with unsuficient photoprotection as well as with heat-induced alterations on the structure, composition, and functional performance. Therefore, these results confirm a notable PSs preservation and a global photochemical functioning in coffee (Rodrigues et al., 2016), thus highlighting that thylakoid membranes function was largely uncompromised. To this would have likely contributed the significant quantitative and qualitative lipid profile adjustments in chloroplast membranes under heat (Scotti-Campos et al., 2019), and the maintenance or reinforcement of the pools of several protective and antioxidative molecules (Martins et al., 2016).

In sharp contrast with the reinforcement up to 37/28°C, RuBisCO and Ru5PK activities suffered the strongest impacts at 42/30°C among the parameters that explore the potential values (*A_max_*, PSs activities), with at least a 65% reduction of their maximum activity values, thus reflecting much stronger impacts than those promoted. In contrast to was found up to 37/28°C, RuBisCO and Ru5PK activities were remarkably affected at 42/30°C to a gretear extent than was On the other hand, RuBisCO and Ru5PK activities were remarkably affected from 37/28°C to 42/30°C to a gretear extent than was by single drought exposure. In fact, RuBisCO was found to be the most heat sensitive component in the photosynthetic machinery of *Coffea* spp. (Rodrigues et al., 2016), which agrees with observations of major negative impacts on its activity over a range of abiotic stresses, with direct negative impacts on crop productivity (Galmés et al., 2013).

### Impact of the Harsh Conditions of Combined Severe Water Deficit and High Temperature on Photosynthetic Functioning

Stress superimposition did not affect either tissue water status or modify the stomatal SD and SA patterns promoted by temperature. However, some interaction was depicted in *P_n_* which tended to even lower values at 42/30°C than under the single exposure to drought (SWD plants at 25/20°C) or heat (WW plants at 42/30°C) in both genotypes. Since *g_s_* was maintained at very low values, similar to those of SWD plants at 25/20°C, this additional *P_n_* reduction has been likely to be related to further non-stomatal impacts which lead *P_n_* to the minimal values observed over the entire experiment. In fact, severe drought may predispose the leaves to photoinhibitory damage given that a strong stomata closure will impose drastic restriction of CO_2_ diffusion into the chloroplast, reducing photochemical energy use and promoting energy overcharge (Baker and Rosenqvist, 2004; Haworth et al., 2018), with the concurrent need to an increased thermal energy dissipation (*Y_(NPQ)_*). However, it seems relevant that in some cases at 42/30°C, the SWD plants displayed better values (*e.g. F_0_*, *Y_(NPQ)_*) than the WW plants, in both genotypes, as well also in *F_v_/F_m_*, and *Y_(NO)_* in Icatu, showing important resilience of these plants to stress combination. In fact, even under the most stressful conditions both genotypes did not show an increased non-regulated energy dissipation in PSII (*Y_(NO)_*), which is known to rise only under harsh environmental conditions (Busch et al., 2009). Instead, *Y_(NPQ)_* reached maximum values, showing that protective mechanisms were still functioning to protect the photosynthetic apparatus from additional damages caused by the excessive excitation (Pompelli et al., 2010; Rodrigues et al., 2016), likely associated with the presence of photoprotective carotenoids (Martins et al., 2016; Ramalho et al., 2018b).

When looking at thylakoid-related photochemical events and components, the exposure to heat aggravated the drought impact (SWD plants) on the PSs activity, electron carriers and the proteins involved in CEF of both genotypes. However, while Icatu showed a negative interaction above 25/20°C, this was not observed in CL153 that showed an impact of drought at 25/20°C but some parameters were mostly insensitive to temperature rise in SWD plants. Electron carriers can be affected under abiotic stress conditions (Nouri et al., 2015) since they are close to the production sites of highly excited molecules of either chlorophyll or oxygen (Logan, 2005). Such oxidative stress conditions can promote the dissociation of the PSII oxygen evolving complex (*OEC*), resulting in greater inhibition of electron transport to the receptor side of PSII (Wang et al., 2018). This was not the case in coffee genotypes, which maintained close PSII activities either including or not the OEC, irrespective of stress conditions (**Figure 3**). In contrast, PSI photoinhibition can be mostly promoted by *ROS* produced on the receptor side of PSI through the Mehler reaction (Sonoike, 2011; Yan et al., 2013). PSI is often considered to be more resistant to photoinhibition through an efficient scavenging of *ROS* produced on the reducing side of PSI (Ozakca, 2013). In any case, under the present experimental conditions, PSI and PSII were affected to a similar extent within each genotype by drought (only CL153), by heat, or even by the stress combination, suggesting that thermal dissipation and/or antioxidative mechanisms protected both PSs indistinctly. In fact, it should be highlighted a high degree of tolerance of SWD plants given that relevant PSs activity and electron carrier contents were preserved under the harshest conditions, even under conditions in which CEF might had a limited role due to PGR5 and NDH proteins reduction at 42/30°C.

Finally, we have demonstrated that the combination of the highest temperature and drought exacerbated the impacts on RuBisCO and Ru5PK activities relative to those promoted by each single stress. In fact, with activity reductions higher than 80%, these enzymes were the most affected photosynthetic components, thus likely limiting the photosynthetic pathway, in good agreement with the minimal *A_max_* values. This limiting point in the coffee acclimation to harsh environmental conditions clearly agreed with the estimates pointing that reductions in RuBisCO activity will be one of the main effects caused by climate change, and should be considered in prediction models on future plant productivity (Galmés et al., 2013). Indeed, given that only a few parameters showed an aggravated status under the imposition of both stresses, we contend that the impacts related to the Calvin-Benson cycle enzymes will play a key role in determining the performance of the photosynthetic apparatus irrespective of genotype.

### Recovery From Stress Exposure and After Effects

Despite the superior performance of Icatu upon drought, and a relatively better performance of CL153 at the highest temperature, some interesting results were obtained along the recovery period after stress relief, with diverse promptness and extent of recoveries of physiological and biochemical parameters.

An almost full recovery of *g_s_* and *Ψ_pd_* was observed in SWD plants from both genotypes, although with a consistent trend to lower water status (*e.g.*, *Ψ_pd_* values in CL153 until Rec14). This suggested a considerable tolerance of the hydraulic system under the harsh conditions of combined water deficit and heat.

However, some marked aftereffects persisted in plants submitted to the combined stresses (SWD plants), usually stronger in CL153, namely in *A_max_*, *Y_(NPQ)_*, *F_v_’/F_m_’*, PSs activity, and *PQ-9* content. In fact, only the SWD CL153 plants were unable to show a full recovery of several parameters (*e.g.*, *P_n_*, *Y_(II)_*, *Y_(NPQ)_*) by Rec14, denoting an exacerbated sensitivity to stress interaction. Also, *F_s_/F_m_’* was kept at high values by Rec14, in line with lower energy driven to photochemical events (*Y_(II)_*) (all plants), and with some higher values of *Y_(NPQ)_* (SWD plants from both genotypes) or *Y_(NO_*_)_ (WW and SWD plants of Icatu).

Several parameters recovered in WW plants of both genotypes (*e.g.*, *A_max_*, *F_v_/F_m_*, *F_v_’/F_m_’*, *q_L_*, *F_s_/F_m_’*, PQ-9 content), but a faster and/or greater recovery was observed in Icatu than in CL153 in WW or SWD plants (*P_n_*, PSs activity, *Cyt f* and *b_563_* contents, Ru5PK activity). This denotes lower impairments upon stress exposure and/or greater recovery capability. Overall, our data agree with previous findings for Icatu resilience involving improved antioxidative mechanisms and adjustments of chloroplast membrane lipids, which ultimately minimize oxidative damages under cold and/or drought (Fortunato et al., 2010; Partelli et al., 2011; Ramalho et al., 2014b; Ramalho et al., 2018b).

It was noteworthy that in a few cases the SWD plants recovered better than their WW counterparts (*e.g.*, PSs and Ru5PK activities, *Cyt b_559LP_* and *b_563_* contents in CL153; and RuBisCO and Ru5PK activities in Icatu), suggesting some degree of stress cross-tolerance related to protecting mechanisms of these photosynthetic components. This was likely related, at least partly, to a more effective *ROS* control, as also observed under the combined exposure to cold and drought (Ramalho et al., 2018b).

Finally, some aftereffects were observed by the end of the experiment, particularly in the plants exposed simultaneously to both stresses, thus justifying the plant response to maintain an increased potential for CEF and thermal dissipation mechanisms. Furthermore, most photosynthetic components recovered between Rec4 and Rec14, suggesting that coffee plants present an interesting resilience to water scarcity and heat, which may help the sustainability of this crop in a scenario of climate variability.

## Conclusions

In the context of ongoing climate changes and extreme weather events, this study thoroughly assessed the impacts of both single and combined drought and heat stressful conditions on the photosynthetic functioning.

Globally, single severe drought significantly affected most gas exchange and fluorescence parameters in both genotypes. This was likely associated with a prevalence of stomata limitations of photosynthesis particularly in Icatu, that showed a better photosynthetic performance and protection of both photosystems, associated with CEF involving PSII and PSI, together with the increase in thermal dissipation mechanisms (the latter in both genotypes). In contrast, in CL153, RuBisCO activities, electron transport rates and Cyt content were reduced, and only minor changes were observed regarding PG5 and NDH proteins. Notably, the reduction of energy use in photochemical events was compensated for by rises in photoprotection and not by uncontrolled energy dissipation in both genotypes, thus reflecting a common triggering of acclimation mechanisms to avoid damages.

A strong photosynthetic heat tolerance was found in WW plants until temperatures well above those considered adequate for the coffee crop. Most parameters related to the photosynthetic potential were barely affected up to 37/28°C or 39/30°C. Notably, CEF around both PSs was also likely involved in the response to heat, particularly in Icatu that showed increased values in all electron carriers, and PG5 and NDH proteins up to 37/28°C. A further increase to 42/30°C impacted most parameters, evidencing that the tolerance threshold in these genotypes was exceeded. At this temperature, *g*_s_ was mostly governed by stomata opening control than by stomatal morphological traits. In any case, the simultaneous reduction in *A_max_* and increase in *C_i_* indicate the prevalence of non-stomatal limitations of photosynthesis, particularly in RuBisCO and Ru5PK. These enzymes were, by far, the most sensitive components, in sharp contrast with PSs activity and electron carrier contents that were mostly unaffected, even with a reduction of PG5 and NDH proteins from 37/28 to 42/30°C.

Stress interaction was largely absent until 37/28°C, with drought being the main constraint until this temperature. However, the two extreme conditions (SWD plants at 42/30°C) aggravated some single stress impacts, with emphasis on the PSI, PSII, and enzymes activities, and electron carriers, the latter somewhat stronger in Icatu (as also in *A_max_*). Noteworthy, even under such harsh conditions uncontrolled energy dissipation did not increase due to reinforcements in energy thermal dissipation.

Strong coffee’s resilience to these stress conditions was observed given that most photosynthetic parameters recovered between the 4^th^ and 14^th^ days after stress relief. However, some aftereffects persisted, mostly in SWD plants (*Y_(NPQ)_*, *F_v_’/F_m_’*, PSs activity, Cyts) by the end of the experiment, justifying the maintenance of a higher potential for protective mechanisms, reflected in energy thermal dissipation and CEF. Among genotypes, Icatu showed a faster and/or greater recovery in WW or SWD plants in several parameters (*P_n_*, *F_v_’/F_m_’*, *q_N_*, PSs and enzymes activities, most electron carriers content) than did CL153.

Overall, genotype-related impacts on the photosynthetic performance were observed under the exposure to the two major environmental constraints, and their interaction. Icatu was more tolerant to drought, and displayed a better recovery after stress relief. Both genotypes were clearly tolerant until 37/28°C, but were deeply affected at 42/30°C, with some additional impacts under the stress superimposition (*e.g.*, RuBisCO activity and electron carriers). The photochemical components were highly tolerant to drought (Icatu), heat and stress interaction (both genotypes), in sharp contrast with enzymes (RuBisCO and Ru5PK) that were highly sensitive, thus, deserving special attention in breeding programs regarding these environmental limiting conditions.

## Data Availability Statement

The mass spectrometry proteomics data have been deposited to the ProteomeXchange Consortium via the PRIDE partner repository with the dataset identifier PXD019474 and DOI: 10.6019/PXD019474 for *C. arabica* proteome, and the dataset identifier PXD019541 and DOI: 10.6019/PXD019541 for *C. canephora* proteome.

## Author Contributions

DD: Investigation, formal analysis, writing (original draft). FL: Conceptualization, Investigation, writing (review and editing), project administration. AR: Investigation, validation, formal analysis. JS: Investigation, formal analysis. IMa: Investigation, validation, formal analysis. DG: Investigation, visualization, formal analysis. JA: Investigation, visualization, formal analysis, supervision. MS: Investigation, validation, formal analysis. SM: Investigation, validation, formal analysis. WR: Investigation, formal analysis, writing (review and editing); MS-C: Investigation, validation, formal analysis; IMo: Investigation, validation. formal analysis; IP: Investigation, validation, formal analysis; PS-C: Investigation, validation; FP: Investigation, supervision; EC: Investigation, supervision, writing (review and editing); AR-B: Conceptualization, investigation, formal analysis, supervision, Funding acquisition;. FD: Conceptualization, Data Analysis, writing (review and editing); JR: Conceptualization, investigation, visualization, data analysis, supervision, project administration, funding acquisition, writing (original draft, review and editing).

## Funding

This work received funding from the European Union’s Horizon 2020 research and innovation program under grant agreement No 727934 (project BreedCAFS), and from national funds from Fundação para a Ciência e a Tecnologia (FCT), Portugal, through the project PTDC/ASP-AGR/31257/2017, and the research units UIDB/00239/2020 (CEF), and UIDP/04035/2020 (GeoBioTec). Funding from Brazil through FAPERJ (grant E-26/202.323/2017, WR) and CNPq (FP, EC, and FD) are also greatly acknowledged.

## Conflict of Interest

The authors declare that the research was conducted in the absence of any commercial or financial relationships that could be construed as a potential conflict of interest.
